# Targeting the 5′ untranslated region of *SMN2* as a therapeutic strategy for spinal muscular atrophy

**DOI:** 10.1016/j.omtn.2020.12.027

**Published:** 2021-01-05

**Authors:** Audrey M. Winkelsas, Christopher Grunseich, George G. Harmison, Katarzyna Chwalenia, Carlo Rinaldi, Suzan M. Hammond, Kory Johnson, Melissa Bowerman, Sukrat Arya, Kevin Talbot, Matthew J. Wood, Kenneth H. Fischbeck

**Affiliations:** 1 Neurogenetics Branch, National Institute of Neurological Disorders and Stroke, National Institutes of Health, Bethesda, MD 20892, USA; 2 Nuffield Department of Medicine, University of Oxford, Oxford OX3 7BN, UK; 3 Department of Paediatrics, University of Oxford, Oxford OX1 3QX, UK; 4 Department of Physiology, Anatomy and Genetics, University of Oxford, Oxford OX1 3QX, UK; 5 Nuffield Department of Clinical Neurosciences, University of Oxford, Oxford OX3 9DU, UK

**Keywords:** antisense oligonucleotides, spinal muscular atrophy, 5′ UTR, SMN2

## Abstract

Spinal muscular atrophy (SMA) is a neuromuscular disorder caused by mutations in the survival motor neuron 1 (*SMN1*) gene. All patients have at least one copy of a paralog, *SMN2*, but a C-to-T transition in this gene results in exon 7 skipping in a majority of transcripts. Approved treatment for SMA involves promoting exon 7 inclusion in the *SMN2* transcript or increasing the amount of full-length SMN by gene replacement with a viral vector. Increasing the pool of *SMN2* transcripts and increasing their translational efficiency can be used to enhance splice correction. We sought to determine whether the 5′ untranslated region (5′ UTR) of *SMN2* contains a repressive feature that can be targeted to increase SMN levels. We found that antisense oligonucleotides (ASOs) complementary to the 5′ end of *SMN2* increase SMN mRNA and protein levels and that this effect is due to inhibition of *SMN2* mRNA decay. Moreover, use of the 5′ UTR ASO in combination with a splice-switching oligonucleotide (SSO) increases SMN levels above those attained with the SSO alone. Our results add to the current understanding of SMN regulation and point toward a new therapeutic target for SMA.

## Graphical Abstract

**Figure:**
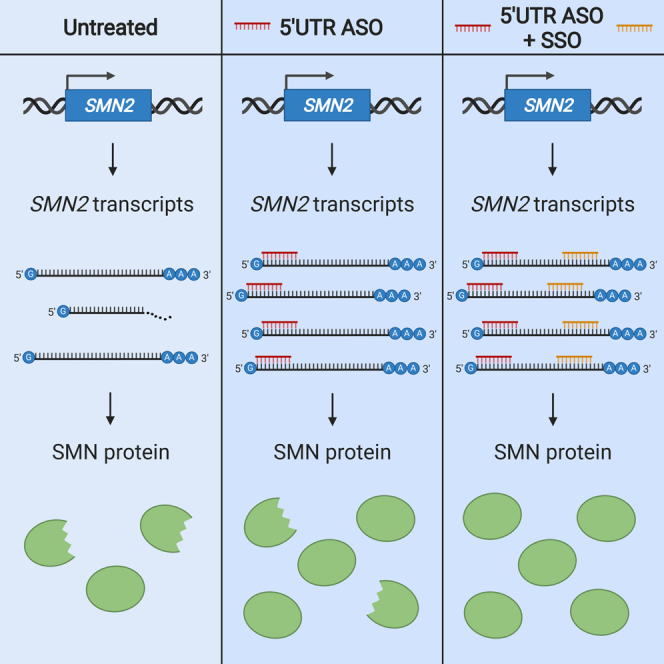

## Teaser

Spinal muscular atrophy (SMA) is caused by SMN protein deficiency. Winkelsas et al. show that antisense oligonucleotides complementary to the 5′ end of *SMN* transcripts increase SMN protein levels by stabilizing *SMN* mRNA. In cells, this strategy can be used in combination with splice-switching oligonucleotides (i.e., nusinersen) for SMA treatment.

## Introduction

Spinal muscular atrophy (SMA) is an autosomal recessive neuromuscular disorder caused by loss-of-function mutations in the survival motor neuron 1 (*SMN1*) gene.^1^ Although *SMN1* is a ubiquitously expressed gene, SMA is primarily a disease of lower motor neurons. Denervation results in symmetrical muscle weakness, often within weeks or months of birth.^2^ *SMN1* encodes the SMN protein, which has a well-characterized function in small nuclear ribonucleoprotein (snRNP) assembly.^3^^,^^4^ Other cellular processes where SMN is likely involved include axonal mRNA transport and local translation and endocytosis, which may account for the motor neuron vulnerability in SMA.^5^, ^6^, ^7^, ^8^

A complete absence of SMN protein results in embryonic lethality.^9^ All SMA patients have at least one copy^10^^,^^11^ of an *SMN1* gene paralog, *SMN2*, which arose from a duplication of the SMN locus on chromosome 5.^1^ *SMN2* does not fully compensate for the loss of *SMN1*; due to a C-to-T transition that results in exon 7 skipping in a majority of transcripts, only 10 percent to 20 percent of *SMN2* mRNAs encode the fully functional SMN protein.^12^, ^13^, ^14^ Clinically, disease severity therefore correlates with *SMN2* copy number and full-length *SMN2* transcript level.^15^^,^^16^

Increasing the level of SMN, via targeting *SMN2* or via gene therapy,^17^ has been a primary therapeutic strategy for SMA. Nusinersen is an antisense oligonucleotide (ASO) that increases the proportion of *SMN2* transcripts containing exon 7.^18^, ^19^, ^20^ Another SMN splice modifier, risdiplam, is a small molecule that has the advantage of being orally bioavailable.^21^^,^^22^ Targeting splicing as a means of increasing SMN levels has a ceiling effect determined by the abundance of *SMN2* transcripts in cells. Increasing the total pool of *SMN2* transcripts and increasing the translational efficiency of these transcripts are two strategies to overcome the ceiling effect associated with the splice-switching strategy.

To identify a new target, we looked in the *SMN2* 5′ untranslated region (5′ UTR). It is known that regulatory motifs within 5′ UTRs influence gene expression by controlling transcript stability, translational efficiency, and subcellular localization.^23^^,^^24^ This occurs through a dynamic interplay between *cis-acting* elements (i.e., the primary sequence and secondary structures of 5′ UTRs) and *trans-acting* factors (i.e., RNA-binding proteins and noncoding RNAs). We sought to determine whether the 5′ UTR of *SMN2* contains a repressive feature that limits its expression, of which targeting could increase SMN levels. We identified sequences in the 5′ UTR that can be targeted with an ASO, which through binding to the 5′-most (ASO #1) end of the *SMN2* transcript, increases SMN levels by stabilizing *SMN2* mRNA. We found that the 5′ UTR ASO used in combination with a splice-switching oligonucleotide (SSO) augments SMN above levels achieved with an SSO alone. Our results add to the current understanding of *SMN2* mRNA turnover and point toward a new therapeutic target for SMA that can be pursued as a combinatorial therapy.

## Results

### Targeting the 5′ end of *SMN2* with ASOs increases SMN protein levels

The 5′ UTR of *SMN2*^25^, ^26^, ^27^ contains a start codon 157 nucleotides upstream of the canonical *SMN* translation initiation codon. Nearly one-half of all human transcripts contains upstream open reading frames (uORFs), features that may attenuate translation of the main protein coding sequence.^28^ Recently, it was shown that ASO binding at start codons in 5′ leader sequences can prevent translation initiation at uORFs and promote translation of primary ORFs.^29^ In addition, ASOs have been used to increase translation of mRNAs containing other types of 5′ UTR inhibitory elements, such as G-quadruplexes or hairpin structures.^30^^,^^31^

**Figure 1:**
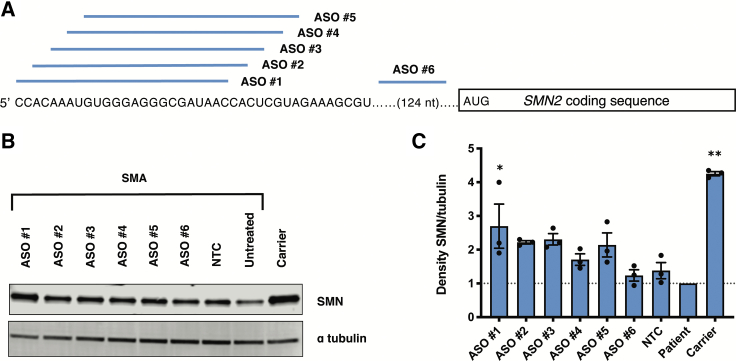
Targeting the 5′ end of *SMN2* with ASOs increases levels of SMN in fibroblasts (A) A schematic depicting the tiling of 2′-OMe ASOs in 2 nt increments along the beginning of the *SMN2* 5′ UTR. (B) Immunoblot (15 μg per lane) showing SMN protein levels in SMN-deficient fibroblasts (GM00232) treated with 600 nM 5′ UTR ASOs or a nontargeting control (NTC) oligo, where indicated. (C) SMN protein levels normalized to alpha tubulin and then calculated as a fold change relative to SMN levels in untreated SMA patient cells (represented by the dotted line). SMN levels from carrier cells are provided for reference. Error bars show SEM. Statistical significance was determined by one-way ANOVA followed by Dunnett’s test in comparison to NTC. n = 3; ∗p = 0.02; ∗∗p < 0.0001.

We designed a series of overlapping 2′-*O*-methyl (2′-OMe) ASOs in 2-nucleotide increments across the 5′ region of the *SMN2* transcript, including the uORF associated with the putative start codon (Figure 1A). ASOs in which all bases contain the 2′-OMe modification operate via steric hindrance rather than RNase H-mediated RNA degradation and can therefore be used to increase gene expression. Table S1 shows the sequence of each ASO. Transfection of ASOs targeting the 5′ UTR in SMA patient-derived fibroblasts resulted in increased SMN protein levels compared to the level of SMN in untransfected patient cells or those treated with a nontargeting control (NTC) ASO (Figures 1B and 1C). Stepping 5′ to 3′ across the UTR, there is a downward trend in the ASO effect on SMN expression, indicating that the critical target region is close to the 5′ terminal cap. We decided to use ASO #1 in the experiments that follow since it demonstrated the largest biological effect, with an average 2.7-fold increase in SMN protein levels. We tested this ASO #1 in a second SMA cell line (Figure S1) to confirm that its effects are not cell-line specific. We found a similar trend using a 5′ UTR phosphorodiamidate morpholino oligomer (PMO) conjugated to a cell-penetrating peptide (pPMO) in motor neuron-like cells chemically differentiated from SMA patient-derived induced pluripotent stem cells (iPSCs; Table S2; Figure S2).^32^^,^^33^

**Figure 2:**
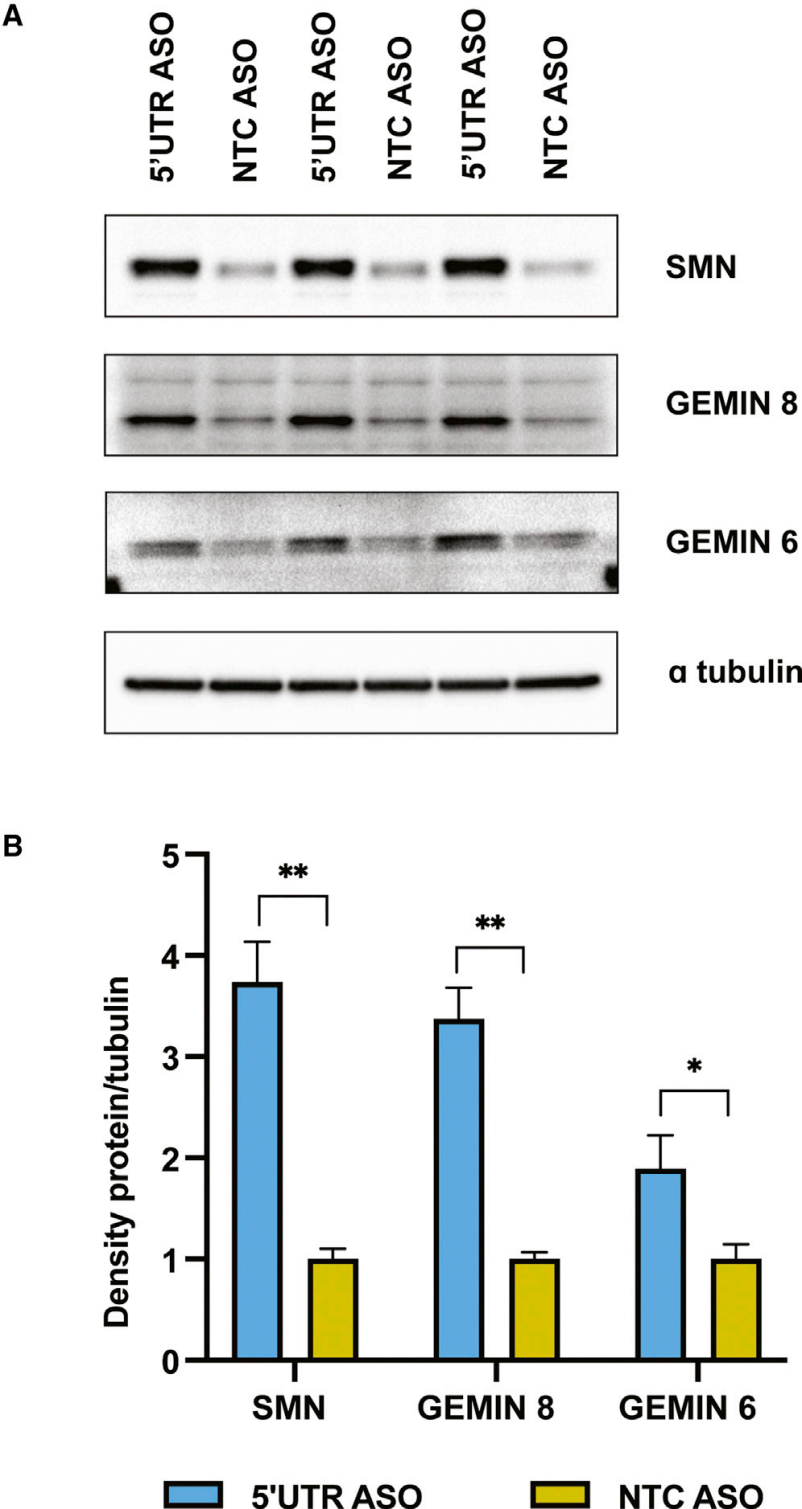
Levels of SMN complex members increase with 5′ UTR ASO treatment (A) Immunoblot showing levels of SMN, Gemin6, and Gemin8 in SMA fibroblasts following transfection with 300 nM ASO. ASOs were fully modified with 2′-MOE bases and phosphorothioate (PS) linkages. 35 μg protein per sample was resolved due to difficulty in detecting Gemin6. (B) Levels of the proteins of interest were normalized to alpha tubulin, and this ratio was then averaged for the two sample groups (5′ UTR ASO and NTC ASO, in triplicate). The graph shows the level of each protein as a fold change relative to protein levels in cells transfected with the NTC ASO. Error bars show propagated error. Statistical significance was determined by t test between the normalized signal intensity values for the two sample groups. n = 3; ∗p < 0.02; ∗∗p < 0.0002.

We also tested an ASO with the same sequence as ASO #1 but with 2′-*O*-(2-methoxyethyl) (2′-MOE)-modified bases. 2′-MOE-modified ASOs are known to undergo less nonspecific protein binding and are among the most widely used in clinical trials.^34^^,^^35^ We found that treating human SMA fibroblasts with the 5′ UTR 2′-MOE increases SMN protein levels 3.7-fold (Figures 2A and 2B). The increased efficacy observed in Figure 2 compared to Figure 1 is likely attributable to the shift from 2′-OMe to 2′-MOE chemistry.

Studies in fibroblasts derived from Taiwanese SMA mice, which are null for mouse *Smn* but contain a 115-kb human DNA sequence containing *SMN2*,^36^ showed no effect of 5′ UTR ASO treatment (Figures S3A and S3B). However, the 5′ UTR ASO is effective in mouse embryonic fibroblasts (MEFs) containing the PAC 215P15-derived human *SMN2* transgene^37^^,^^38^ (Figures S3C and S3D). This could be due to differences in the background genetics of the two mouse strains.

The SMN protein is part of a large protein complex where it associates with Gemin proteins. Previous investigation demonstrated that levels of Gemin6 and Gemin8, which are core components of the SMN complex, correlate with SMN expression levels.^39^ After seeing the effect of the ASO on SMN levels, we sought to determine whether Gemin levels are also increased. If so, this would indicate functional correction of the SMN deficiency with ASO treatment. Indeed, by immunoblot, we found the increase in SMN to be accompanied by 1.9- and 3.4-fold increases in Gemin6 and Gemin8, respectively (Figures 2A and 2B).

### The 5′ UTR ASO increases *SMN2* mRNA levels in fibroblasts by stabilizing transcripts

**Figure 3:**
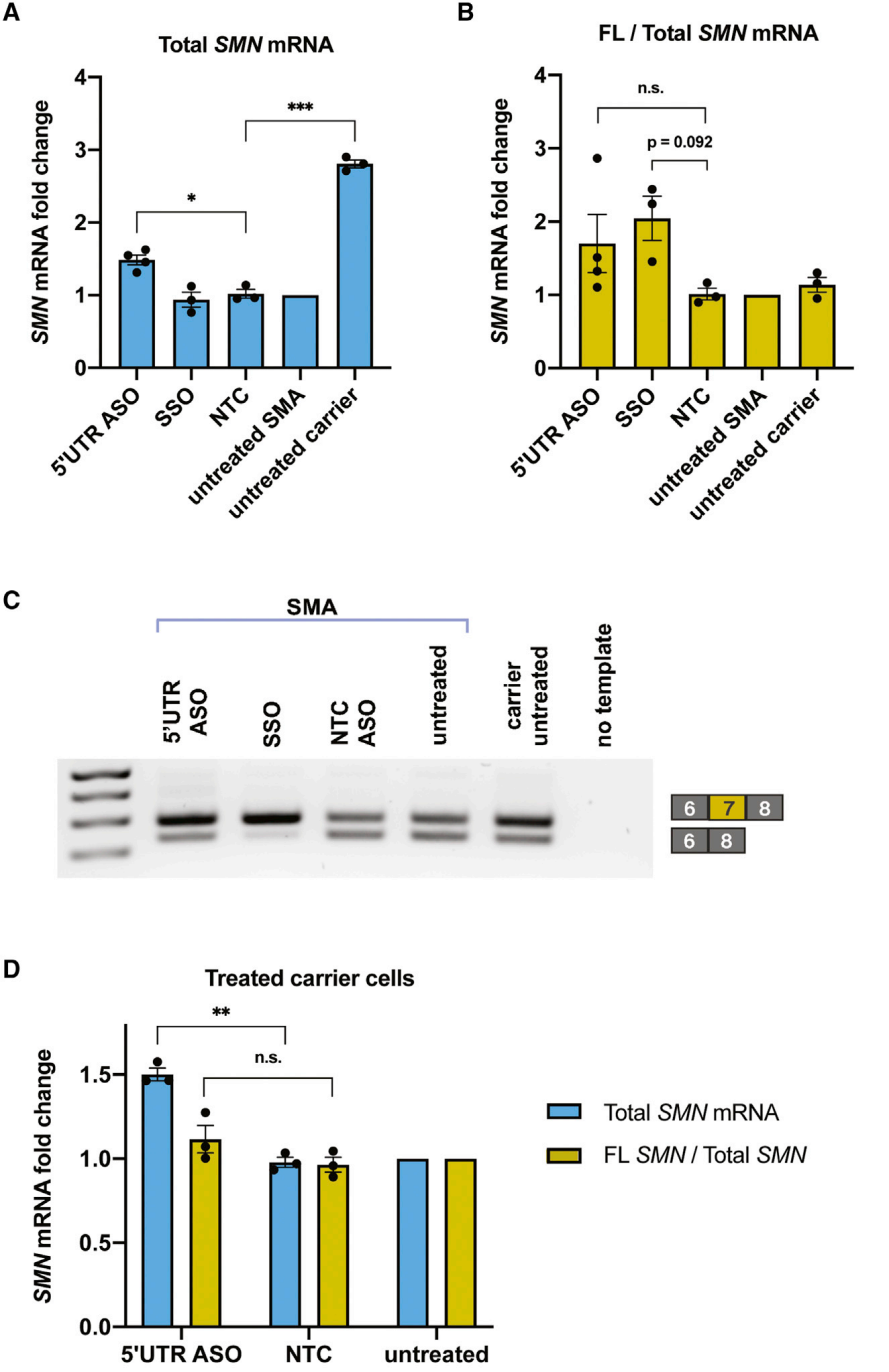
An ASO targeting the 5′ end of *SMN2* increases *SMN* mRNA levels (A) SMA fibroblasts were transfected with 600 nM 2′-OMe 5′ UTR ASO, splice-switching oligonucleotide (SSO), or NTC ASO. qRT-PCR measured total *SMN* mRNA levels. Expression was normalized to *GAPDH* and calculated as a fold change relative to levels in untreated SMA cells. (B) qRT-PCR analysis of the ratio of full-length (FL) *SMN* to total *SMN* transcript levels, measured with primers overlapping exon 7 or primers spanning the exon 2a-2b junction, respectively. (C) RT-PCR to qualitatively visualize alternative splicing with 600 nM ASO treatment. The amplicon from the full-length isoform is 292 bp, whereas the amplicon from the Δ7 isoform is 238 bp. (D) As in (A) and (B), levels of total *SMN* mRNA or the ratio of full-length to total *SMN* mRNA were measured via qRT-PCR. Data in this panel are from ASO treatment in fibroblasts from a carrier of SMA (1 copy *SMN1*, 5 copies *SMN2*). Expression was normalized to *GAPDH* and calculated as a fold change relative to levels in untreated SMA cells. Error bars show SEM. Statistical significance was determined by one-way ANOVA followed by Dunnett’s test in comparison to NTC. n = 3/4; ∗p < 0.005; ∗∗p < 0.001; ∗∗∗p < 0.0001; n.s., not significant.

We next performed qRT-PCR to determine whether *SMN2* transcript levels increase following ASO treatment. Compared to untreated cells or cells treated with a NTC ASO, the 5′ UTR ASO increases total *SMN* mRNA levels in both SMA patient fibroblasts and carrier fibroblasts (Figures 3A and 3D). Total *SMN* mRNA levels were measured with primers spanning the exon 2a-2b junction and are thus irrespective of exon 7 inclusion.

Transcripts containing exon 7 are the most therapeutically relevant, as they encode the full-length SMN protein. This prompted us to check levels of the full-length and Δ7 *SMN* isoforms by RT-PCR and by qRT-PCR. To our surprise, the 5′ UTR ASO led to a shift toward a full-length transcript (with exon 7 inclusion) in patient fibroblasts (Figures 3B and 3C). In carrier fibroblasts from an unaffected individual with higher baseline SMN levels, the ASO increases steady-state mRNA without affecting the ratio of full-length to total transcripts (Figure 3D). Thus, we suspect that the increased level of total *SMN* mRNA is a direct effect of the 5′ UTR ASO, whereas increased exon 7 inclusion is more likely due to SMN feedback^40^^,^^41^ (caused by an increased pool of snRNPs) in states of SMN deficiency rather than a primary mechanism of action of the ASO.

**Figure 4:**
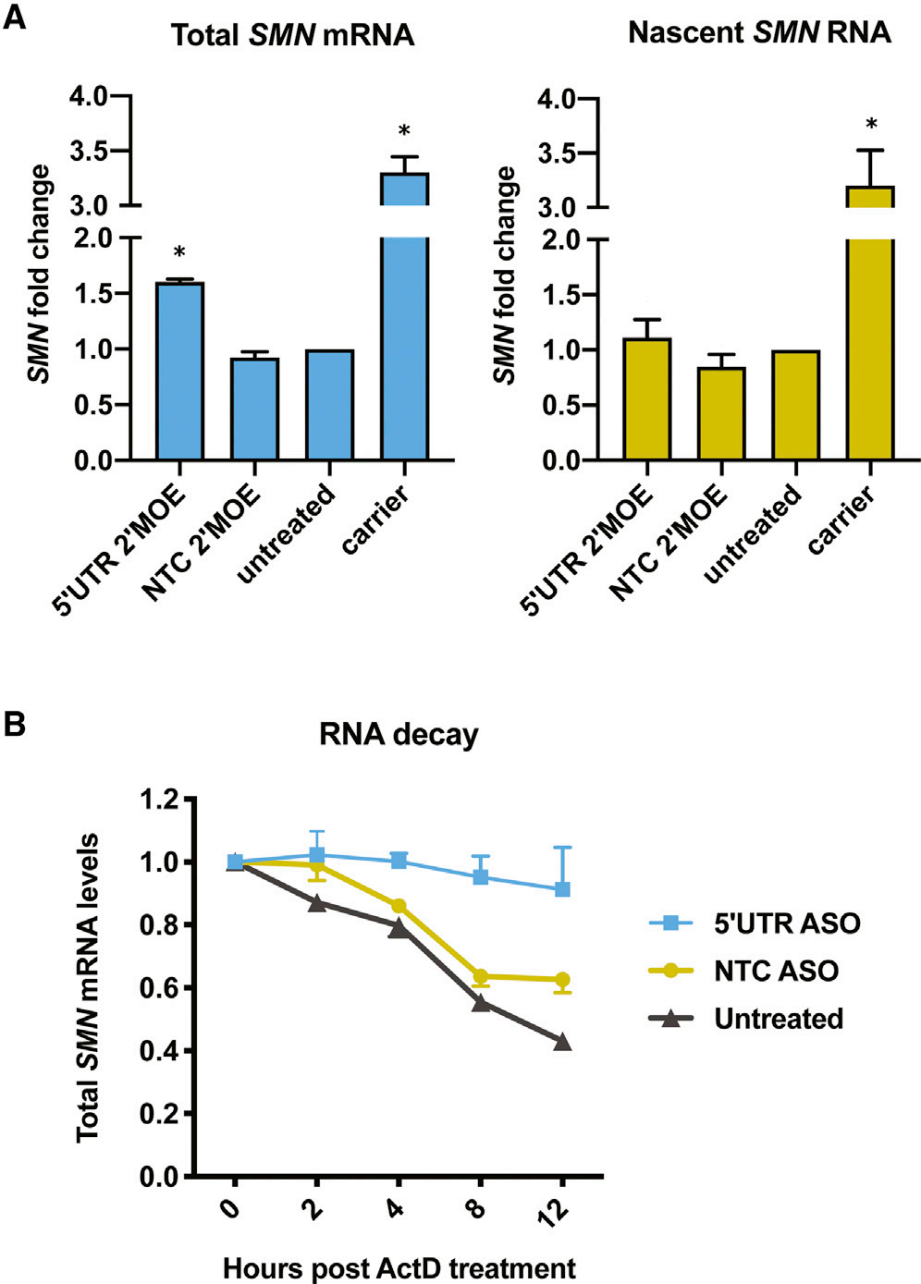
The ASO targeting the 5′ end of *SMN2* increases the level of steady-state *SMN* mRNA by decreasing its turnover (A) SMA fibroblasts were transfected with 150 nM 2′-MOE 5′ UTR ASO or NTC ASO and pulsed with EU. qRT-PCR measured steady-state (total) *SMN* mRNA levels or nascent (biotinylated) *SMN* RNA levels. Expression was normalized to *GAPDH* and compared to levels in untreated SMA cells. Statistical significance was determined by one-way ANOVA followed by Dunnett’s test in comparison to the NTC sample in its group. n = 3; ∗p ≤ 0.007. (B) 48 h post-transfection with 600 nM 2′-OMe ASOs, SMA fibroblasts were treated with actinomycin D (ActD) and collected in TRIzol at the specified time points. qRT-PCR measured total SMN mRNA. n = 3. Statistical analysis was performed using a linear mixed model as described in Materials and methods. The interaction between group and time was significant (χ^2^(2) = 29.2, p value < 0.001). The pairwise differences in slope are as follows: 5′ UTR ASO – NTC ASO = 0.028 (standard error = 0.006, p < 0.001); 5′ UTR ASO – untreated = 0.039 (standard error = 0.006, p < 0.001); NTC ASO – untreated = 0.011 (standard error = 0.006, not significant).

A higher steady-state level of mRNA could either be due to an increased rate of transcription or to a decreased rate of RNA decay. To distinguish between the two possibilities, we pulsed cells with a uridine analog, 5-ethynyl uridine (EU), to measure newly transcribed *SMN2*. Biotinylating the EU allowed us to isolate and quantify only those RNAs transcribed during the 1-h pulse via qRT-PCR. We confirmed assay specificity by blocking transcription with actinomycin D and observing an increase in cycle threshold (C_t_) values of three to nine cycles for *GAPDH* and *SMN*, indicating decreased transcript levels (Table S5). As a positive control, we used SMA carrier fibroblasts that have one copy of *SMN1* and five copies of *SMN2* and thus should transcribe more *SMN* than our patient fibroblasts, which contain two copies of *SMN2* and no *SMN1*. With this RNA-labeling method, we saw no significant difference in nascent *SMN2* transcript levels among cells treated with the 5′ UTR ASO, cells treated with the NTC ASO, or untreated patient cells (Figure 4A). This indicates that the higher steady-state level of *SMN2* is not due to increased transcription but is due instead to slower RNA turnover. Indeed, by treating cells with actinomycin D and collecting RNA at different time points, we found that *SMN2* transcripts are significantly more stable in cells treated with the 5′ UTR ASO (Figure 4B).

### The *SMN2* uORF is not readily translated and does not reduce SMN levels

**Figure 5:**
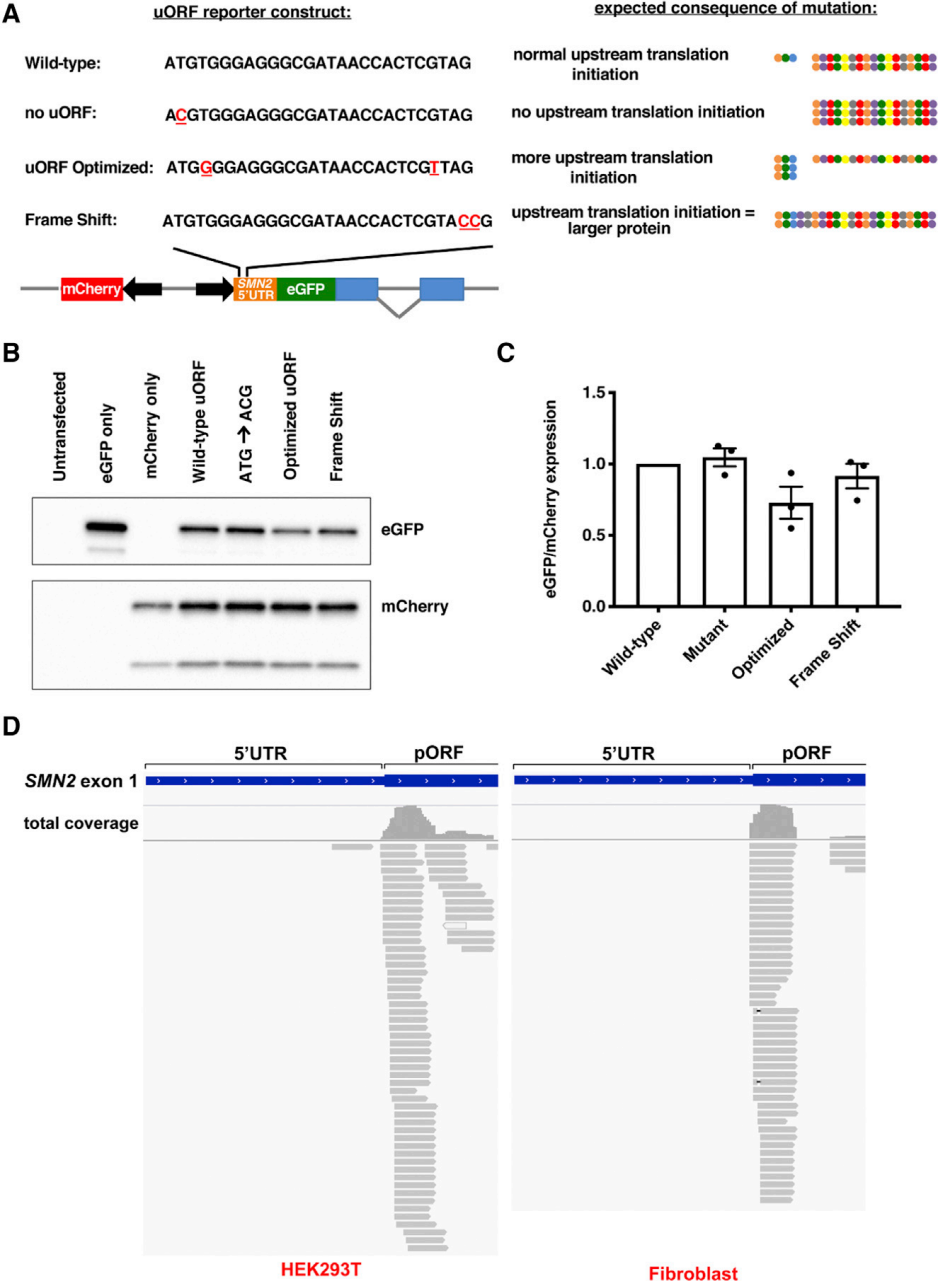
The *SMN2* uORF is not readily translated and does not reduce expression of the primary ORF (pORF) (A) A schematic detailing reporter construct designs, with mutations underlined in red. Expected sizes and relative expression levels of protein products are indicated on the right. The uORF-encoded peptide is represented as the short string (3 circles), whereas the pORF-encoded peptide is represented as the longer string (17 circles). The protein encoded by the frameshift reporter (when translation initiates at the upstream start codon and continues through the 5′ UTR and the pORF) is represented as the longest, continuous string. (B) HEK293Ts were transfected with plasmids and expression levels determined by western blot. 7.5 μg protein was resolved per lane. (C) EGFP protein levels were normalized to mCherry levels and then normalized to expression from cells transfected with the wild-type plasmid. Error bars represent SEM. (D) Ribosome profiling data (HEK293T = GEO: GSM3566399; fibroblast = GEO: GSM1047585) aligned to the *SMN2* locus and visualized using IGV. Gray lines represent individual sequencing reads, with arrows indicating read direction. Total coverage at a particular locus is indicated above reads.

When present, a uORF stop codon may be processed like a premature termination codon (PTC), subjecting the mRNA to nonsense-mediated decay. ASO-mediated inhibition of the uORF could thus explain the increased *SMN2* transcript stability. To better understand the mechanism of action of the 5′ UTR ASO, we designed reporter constructs to study the effects of the uORF on SMN levels. In addition to a construct with enhanced green fluorescent protein (EGFP) under the control of the wild-type *SMN2* 5′ UTR, constructs were made with the following: (1) a mutation to remove the uORF start codon, (2) a mutation that strengthens the sequence context (Kozak sequence) surrounding the uORF start codon, and (3) a frameshift mutation that extends the uORF coding sequence and places it in-frame with GFP (Figure 5A). The latter reporter was designed in order to be able to observe uORF translation initiation, since the uORF peptide is too small to detect by standard techniques.

The expression of these reporters in HEK293T cells showed that removing the upstream start codon (mutating ATG to ACG) does not increase EGFP levels (Figures 5B and 5C), suggesting that the uORF does not have a significant influence on gene expression. There is, however, a decreased EGFP signal when a guanine is present in the +4 position (“uORF optimized” reporter). We can infer from this that the nonoptimized, native uORF is not functional. Finally, the larger protein encoded by the “frame shift” reporter was only detectable at very low levels, indicating that ribosomes do not often engage the uORF (Figure S4).

To rule out a lack of uORF effect being due to an artifact of the reporter system (e.g., use of a nonendogenous transcription start site), we aligned publicly available ribosome profiling data^42^^,^^43^ to the *SMN2* locus (Figure 5D). Sequencing reads corresponding to *SMN1* and/or *SMN2* cannot be accurately mapped to standard reference genomes because multi-mapping reads are discarded or randomly distributed between the paralogs. Thus, we used a custom reference genome in which only the *SMN2* sequence is present.^44^ In the ribosome profiling data, the absence of ribosome-protected fragments mapping to the *SMN2* uORF was in line with our reporter assay findings and supports the conclusion that the uORF in *SMN2* is not a meaningful regulator of *SMN2* expression in fibroblasts or in HEK293T cells.

### A combinatorial therapeutic approach further increases levels of the SMN protein

**Figure 6:**
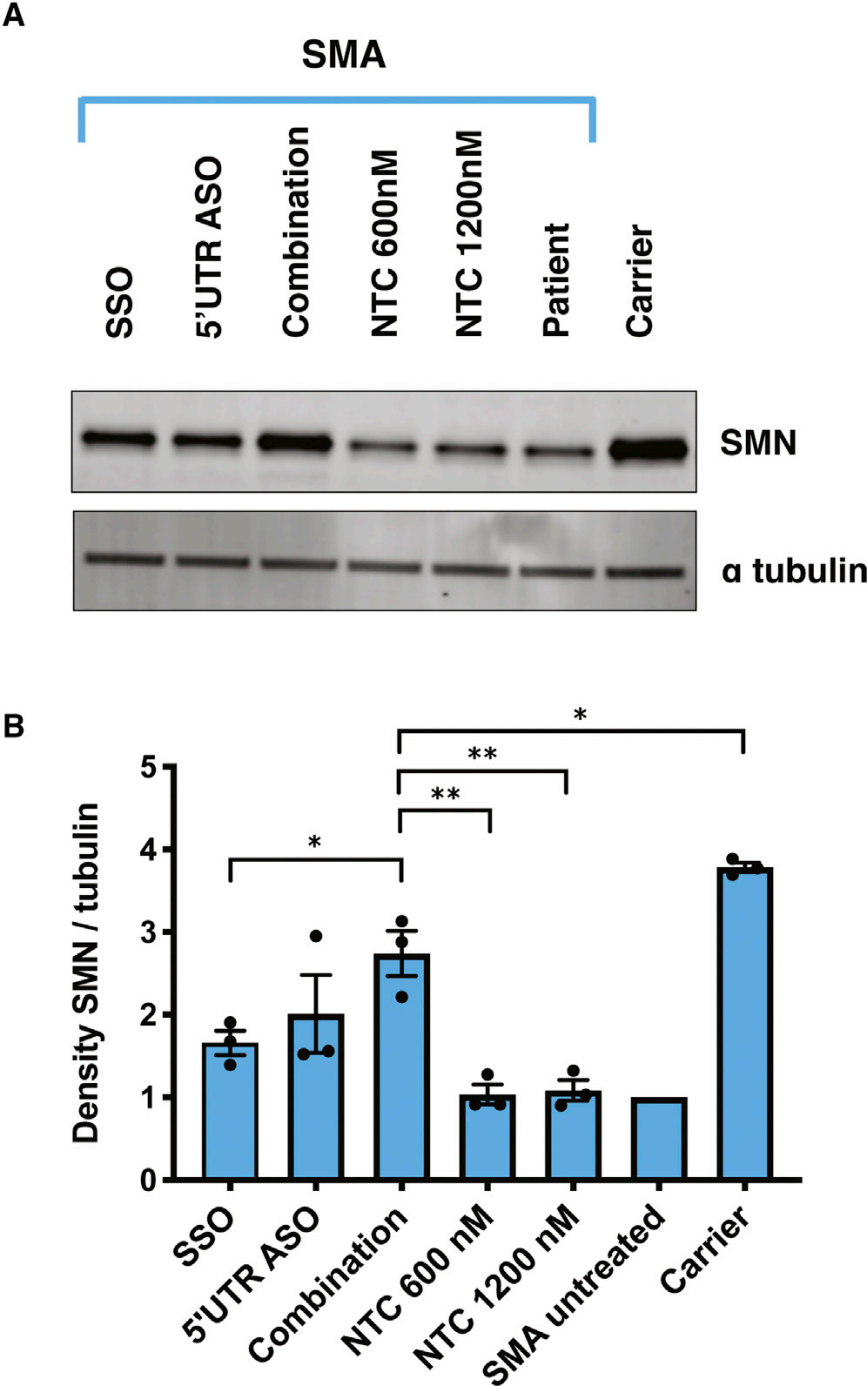
Use of a 5′ UTR ASO in combination with a SSO increases SMN protein levels more than use of the SSO alone (A) SMA fibroblasts were transfected with the 2′-OMe 5′ UTR ASO (600 nM), the 2′-OMe SSO (600 nM), a combination of the two (1,200 nM total), or the 2′-OMe NTC ASO. 15 μg of protein was resolved per lane. (B) SMN levels were normalized to alpha tubulin, and expression was compared to levels in untreated SMA cells. Error bars show SEM. Statistical significance was determined by one-way ANOVA followed by Dunnett’s test in comparison to combination. n = 3; ∗p < 0.05; ∗∗p < 0.001.

Novel strategies to complement splice modulation of *SMN2* may be especially useful for those with SMA who have low *SMN2* copy numbers. The SSO and the 5′ UTR ASO were designed to target distinct RNA processes, leading us to investigate whether a combination of these two ASOs overcomes the ceiling effect associated with the SSO. We tested the 5′ UTR ASO and a SSO that targets the ISSN1 sequence in *SMN2*^20^ separately and jointly. We found that concurrent use of the two ASOs in SMA patient fibroblasts increases SMN protein levels significantly more than use of the SSO alone (Figures 6A and 6B). We speculate that the combined treatment is not significantly different from the 5′ UTR ASO alone because of the shift toward the full-length transcript seen with 5′ UTR ASO treatment (depicted in Figure 3C).

## Discussion

Previously, histone deacetylase (HDAC) inhibitors were shown to increase *SMN2* levels.^45^, ^46^, ^47^ HDAC inhibitors are not specific to the *SMN2* gene, however, and transcriptionally activate a broad array of genes. Some of these nonspecific changes in gene expression may benefit the SMA phenotype, but other changes may be harmful.^48^^,^^49^ Thus, a method for increasing SMN production in a more specific way that complements the splice-switching approach is therapeutically desirable. Here, we show that an ASO, in three different chemistries, with sequence complementary to the 5′ UTR of *SMN2*, increases SMN mRNA and protein levels in human fibroblasts and motor neuron-like cells. Based on our experiments in MEFs, future preclinical studies should use the Burghes and colleagues’^50^ SMNdelta7 SMA mouse model.

In addition to the SMN protein, we show that levels of at least two SMN-associated proteins (Gemin6 and Gemin8) increase with ASO treatment. This is likely because as SMN levels increase, there are more SMN complexes to which Gemin6 and Gemin8 can bind, and this confers stability. Details about the stoichiometry of proteins in the SMN complex are unknown. The 5′ UTR ASO may thus be useful in future experiments to study how proteins such as Gemin6 and Gemin8 are incorporated into the SMN complex, as well as in studying other pathways in which the SMN protein is involved more generally.

Our initial hypothesis was that by blocking translation of the uORF, the 5′ UTR ASO promotes translation of the primary ORF. However, with multiple techniques, we found that the *SMN2* uORF is not normally translated. This may be due to the short distance between the 5′ cap and the uORF start codon (7 nucleotides) or to the weak sequence context surrounding the start codon (T at the +4 position).

Instead, the 5′ UTR ASO stabilizes *SMN2* mRNA. Although it has been established that the SMN protein is degraded by the E3 ubiquitin ligase mind bomb 1 and the proteasome, the process through which *SMN* transcripts are degraded is less clear.^51^ A high-throughput screen identified a quinazoline compound that inhibits the mRNA decapping enzyme DcpS and increases *SMN2* promoter activity in cell-based assays.^52^ Follow-up studies found that this small molecule increases survival and motor function in SMA mice.^53^, ^54^, ^55^, ^56^ Whereas it is known that DcpS hydrolyzes cap structures from mRNA fragments that are generated by extensive 3′ to 5′ exonuclease decay, the specific mechanism through which the quinazoline compound increases *SMN2* expression is unknown.

The idea that the 5′ UTR ASO operates via a mechanism related to decapping is compelling since the ASO is complementary to the *SMN2* sequence immediately adjacent to the 5′ cap. However, we did not find a significant difference in the ASO’s ability to upregulate SMN levels in cells in which decapping factors were knocked down (Figure S5). We cannot rule out this mechanism of action entirely since it is possible that with the knockdown of individual enzymes, there is compensation by other RNA decay machinery.

We found that with the increase in *SMN* mRNA levels, there is a trend toward an increase in the ratio of full-length to exon 7-excluded transcripts in patient fibroblasts. Other compounds that increase levels of SMN have been shown to increase exon 7 inclusion, including an HDAC inhibitor and an ASO that knocks down the antisense strand of *SMN* (called *SMN-AS1*).^57^^,^^58^ Unlike these two compounds, the 5′ UTR ASO works by a transcription-independent mechanism, and we propose that the shift in splicing is due to SMN autoregulation.^40^^,^^41^ The SMN complex is required for spliceosome biogenesis, and it is possible that the transcriptomic changes accompanying an increase in SMN protein levels include modulation of its own alternative splicing. This hypothesis is supported by our observation that there is no change in exon 7 inclusion in carrier fibroblasts treated with the 5′ UTR ASO, where baseline SMN levels (and spliceosome levels) are not low enough to perturb splicing.

Two papers have been published describing the antisense transcript, *SMN-AS1*.^58^^,^^59^ The long noncoding RNA *SMN-AS1* is transcribed from *SMN* intron 1 but binds directly to the *SMN* transcription start site region. Here, it recruits PRC2 and reduces transcription of *SMN2*. Due to the proximity in binding locales, we wondered whether the 5′ UTR ASO reduces *SMN-AS1* activity. However, we found no difference in the transcription rate of *SMN2* with ASO treatment, indicating that the mechanism of action is likely independent of *SMN-AS1*.

Experiments are ongoing to elucidate the mechanism of action of the 5′ UTR ASO. We cannot yet rule out the possibility that the increase in SMN levels is due to off-target engagement, such as electrostatic interactions or sequence-specific interactions between the ASO and proteins. The determination of the mechanism of action of the 5′ UTR ASO may reveal that there are other genes for which expression can be increased using a similar strategy. For example, the upregulation of utrophin via its 5′ UTR may be used for the treatment of Duchenne muscular dystrophy.^60^ For now, our results add to the current understanding of SMN regulation and point toward a new therapeutic target for SMA.

## Materials and methods

### ASO synthesis

Three types of ASO were used in this study: (1) fully modified with 2′-OMe bases and phosphorothioate linkages; (2) fully modified with 2′-MOE bases and phosphorothioate linkages; and (3) PMOs. All 2′-OMe and 2′-MOE ASOs were purchased from Integrated DNA Technologies (IDT). PMOs were purchased from Gene Tools and subsequently conjugated to Pip9b2 as described previously.^61^^,^^62^^,^^65^ ASO sequences are provided in Table S1.

### Cell culture

SMA patient (Coriell; GM00232 and GM03813) and carrier (Coriell; GM03814) fibroblasts were cultured in Dulbecco’s modified Eagle’s medium (DMEM) supplemented with 15% fetal bovine serum (FBS) and maintained in a 37°C incubator with 5% CO_2_. Fibroblast transfections were performed using RNAiMAX transfection reagent (Invitrogen). For each well of a 6-well plate, ASOs or small interfering RNA (siRNA) were complexed with 7.5 μL RNAiMAX in 300 μL Opti-MEM and added to cells at 70% confluency. The next day, media were changed to remove transfection reagents, and cells were harvested 2 days post-transfection (unless stated otherwise in the figure legends). The siRNAs used in these transfections were the following: DCP2 (Dharmacon; 167227), DCPS (Dharmacon; 28960), DXO (Dharmacon; 1797), NUDT3 s22028 (Thermo Fisher Scientific), NUDT16 (Dharmacon; 131870), SMN1/SMN2 (Thermo Fisher Scientific; s446415), XRN2 (Thermo Fisher Scientific; s22412), Negative Control No. 1 (Thermo Fisher Scientific; 4390843), and siGENOME Non-Targeting siRNA #2 (Dharmacon; D-001210-02-05).

HEK293T cells (ATCC) were cultured in DMEM, supplemented with 10% FBS, and maintained in a 37°C incubator with 5% CO_2_. Plasmids were transfected using Lipofectamine 3000 (Invitrogen). Each well of a 6-well plate was transfected with 1 μg DNA, complexed with 3.75 μL Lipofectamine 3000 reagent and 4 μL P3000 reagent in 250 μL Opti-MEM. Media were changed 24 h later, and cells were harvested 2 days after transfection.

iPSCs were derived from fibroblasts grown from skin biopsies collected from type 2 or 3 SMA patients attending the Oxford Motor Neuron Disorders Clinic (under ethical approval granted by the South Wales Research Ethics Committee; ref 12/WA/0186). These were reprogrammed in the James Martin Stem Cell Facility, University of Oxford, using the method indicated in Table S2. Type I SMA iPSCs were a gift of Dr. Jeroen Pasterkamp, University of Utrecht. iPSCs were differentiated into motor neuron-like cells as described previously.^32^^,^^33^ Briefly, the iPSCs were grown on Matrigel. They were then induced using equal volumes of DMEM/F12 and neurobasal medias supplemented with N2, B27, ascorbic acid (0.5 μM), 2-mercaptoethanol (50 μM), compound C (1 μM), and Chir99021 (3 μM). After 4 days in culture, media were further supplemented with retinoic acid (1 μM) and smoothened agonist (500 nM). The following day, the media were changed to media without compound C and Chir99021. The cells were then cultured for 4–5 additional days before being split 1:3 using Accutase. Rock inhibitor was added for 24 h. After splitting, the media were supplemented with growth factors brain-derived neurotrophic factor (BDNF; 10 μM), glial cell line-derived neurotrophic factor (GDNF; 10 μM), N-[N-(3,5-difluorophenacetyl)-L-ala-nyl]-S-phenylglycine t-butyl ester (DAPT) (10 mM), and laminin (0.5 mg/mL) for 7 days. DAPT and laminin were then removed from the media, and the neurons remained in culture until day 28. Neurons were treated with ASOs (with no transfection reagents) on day 24 and again on day 26, before collection on day 28.

### Cloning

To test the effect of the upstream start codon on gene expression, reporter constructs were created using the pBI-CMV4 bidirectional promoter vector (Takara Bio). The plasmid backbone was double digested with BglII and EcoRI and gel extracted using the NucleoSpin Gel and PCR Clean-Up Kit (Takara Bio). Then, DsRed2 was replaced with PCR-amplified mCherry using the In-Fusion HD Cloning Plus Kit (Takara Bio). Mini-prepped mCherry plasmid was double digested with BamHI and NotI for insertion of the reporter protein coding sequence. As a template for the reporter, we used a gBlocks Gene Fragment (IDT) containing the 5′ UTR of *SMN2* followed by the coding sequence of EGFP followed by exon 2 through exon 3 of the human beta-globin gene (*HBB*). The well-characterized *HBB* exon 2, intron 2, exon 3 splice junction was included to make the reporters more sensitive to endogenous gene regulation. This feature is important since one of the means through which uORFs downregulate gene expression is promoting nonsense-mediated decay, a process that requires the presence of an exon junction complex. The gBlocks Gene Fragment was PCR amplified using CloneAmp HiFi PCR Premix (Takara Bio) and 500 nM each primer, where the forward primer contained the desired uORF mutation(s). Primer sequences are provided in Table S7. One of the reporters (frame shift) required a second gBlocks Gene Fragment in order to obtain a plasmid with the desired mutation (Table S6). These PCR products and digested plasmids were gel extracted and cloned using the In-Fusion HD Cloning Plus Kit (Takara Bio), as above.

For all cloning work, One Shot TOP10 Chemically Competent *E. coli* (Invitrogen) was transformed with In-Fusion reaction products. Plasmids were extracted from bacterial cultures using the QIAprep Spin Miniprep Kit (QIAGEN) and tested by restriction enzyme digest. Selected clones were expanded and plasmids extracted using the HiSpeed Plasmid Maxi Kit (QIAGEN). The sequences of inserted DNA fragments were verified in all plasmids by Sanger sequencing (GENEWIZ). Details of primers used for sequencing are provided in Table S8.

### Immunoblotting

Lysates were prepared in radioimmunoprecipitation assay (RIPA) buffer (50 mM Tris-HCl, pH 8.0, 150 mM NaCl, 5 mM EDTA, 1% NP-40 [IGEPAL], 1% sodium deoxycholate, 0.1% sodium dodecyl sulfate [SDS]), supplemented with Halt Protease and Phosphatase Inhibitor Cocktail (Thermo Fisher Scientific) or cOmplete Protease Inhibitor Cocktail (Roche). After lysing on ice, samples were centrifuged (15 min, 14,000 × *g*, 4°C) to remove pelleted material. Protein concentrations were determined by the Bradford assay using Protein Assay Dye Reagent Concentrate (Bio-Rad). Samples were then prepared in RIPA and 4× sample loading buffer (H_2_O, Tris-HCl, 40% glycerol, 0.08 g/mL SDS, 5% [v/v] 2-mercaptoethanol, bromophenol blue). Proteins were resolved on Novex 4%–20% Tris-Glycine WedgeWell Gels (Invitrogen) and transferred to a 0.45-μm polyvinylidene fluoride (PVDF) membrane. The amount of protein loaded per lane in micrograms is indicated in the relevant figure legend. Membranes were blocked with 5% (w/v) milk in Tris-buffered saline and 0.1% Tween (TBST) before incubation with primary antibodies at the indicated dilutions: mouse anti-SMN (BD Biosciences; 610647, 1:1,000 dilution), anti-Gemin6 (Abcam; ab88290, 1:500 dilution), anti-Gemin8 (Abcam; ab46778, 1:1,000 dilution), rabbit anti-alpha tubulin (Abcam, ab4074, 1:5,000 dilution; or Cell Signaling Technology, 2144, 1:2,000), rabbit anti-heat shock protein (HSP)90 (Cell Signaling Technology; 4874, 1:5,000 dilution), rabbit anti-GFP (Abcam; ab290, 1:10,000 dilution), and mouse anti-mCherry (Abcam; ab125096, 1:2,000 dilution). The membranes were then incubated with either IRDye or horseradish peroxidase (HRP) secondary antibodies and detected on a LI-COR Odyssey or a Bio-Rad ChemiDoc XRS+ imaging system, respectively. Primary antibodies and secondary antibodies were incubated overnight at 4°C or 1 h at room temperature with shaking and were followed by three washes with TBST. Densitometric analysis of protein signal was done using ImageJ software.

### RNA stability assay

For mRNA stability assays, fibroblasts were transfected with 600 nM 2′-OMe ASOs as described above. 2 days post-transfection, fibroblasts were treated with media containing 5 μg/mL actinomycin D (Sigma-Aldrich). The cells were then collected in 0.5 mL TRIzol at the indicated time points after treatment with actinomycin D. After adding 100 μL chloroform, the samples were vortexed and centrifuged (15 min, 12,000 × *g*, 4°C). The supernatant was transferred to a new tube, to which 1.5 volumes of 100% ethanol were added. The samples were then pipetted into columns, and RNA purification continued according to the miRNeasy Mini Kit manual (QIAGEN). Total RNA was converted into cDNA using the High-Capacity cDNA Reverse Transcription Kit (Applied Biosystems). qRT-PCRs were performed in triplicate using the QuantStudio 6 Flex Real-Time PCR System (Applied Biosystems). 10 μL qRT-PCR reactions contained Power SYBR Green (Applied Biosystems) with 200 nM of each target primer and cDNA diluted in nuclease-free water (to a concentration for which primers have 90%–110% efficiency). Primer sequences are provided in Table S3.

### Transcription assay

Transcription was measured using the Click-iT Nascent RNA Capture Kit (Invitrogen). In summary, 3 days after ASO transfection, SMA fibroblasts were pulsed with 0.5 mM EU for 1 h and then collected in 0.5 mL TRIzol. 100 μL chloroform was added to each sample, which was then vortexed and centrifuged (15 min, 12,000 × *g*, 4°C). The supernatant was transferred to a new tube, and 1.5 volumes of 100% ethanol were added. The samples were then pipetted into columns, and RNA purification continued according to the RNeasy Mini Kit manual (QIAGEN).

750 ng purified RNA was used in each Click reaction. Subsequently, 400 ng biotinylated RNA was mixed with 25 μL magnetic bead suspension for each binding reaction. After washing away unbound RNA, on-bead cDNA synthesis was performed using the SuperScript VILO cDNA Synthesis Kit (Thermo Fisher Scientific) in a 50-μL final reaction volume. 500 ng total RNA (pre-biotinylation) was also converted to cDNA in a 15-μL reaction using the SuperScript VILO cDNA Synthesis Kit, which represents steady-state mRNA.

qRT-PCRs were performed as 20 μL reactions in triplicate. Reactions contained Power SYBR Green (Applied Biosystems), 200 nM of each target primer, 1 μL cDNA, and nuclease-free water. cDNA of total RNA was diluted 1:4 in nuclease-free water, whereas cDNA of biotinylated RNA was undiluted. qRT-PCR plates were run on the StepOnePlus Real-Time PCR System (Applied Biosystems).

### Splice isoform analysis

*SMN2* exon 7 splicing was qualitatively assessed by gel electrophoresis of RT-PCR products. First, RNA was extracted, and cDNA was converted according to the protocol described above for the RNA stability assay. The cDNA from ASO-treated samples was used as template for PCR. The reaction was performed with PCR Master (Roche) and with primers situated in exon 5 and exon 8 of *SMN2*. Primer sequences are provided in Table S4. Amplicons were resolved on 2% agarose gels. To ensure that the full-length amplicon did not sequester the Δ7 amplicon, a test run was performed in which samples were diluted, denatured by heating to 95°C for 5 min, and then immediately placed on ice until being loaded on a gel. The results were consistent between heated and unheated samples, so the extra denaturing and steps were omitted. Exon 7 splicing was quantitatively assessed for the same samples using the qRT-PCR protocol described for the RNA stability assay.

### Bioinformatics

Ribosome profiling sequencing data from GEO sample accessions (GEO: GSM1047584, GSM1047585, and GSM3566399) were downloaded in the fastq format using the fastq-dump command supported in the NCBI SRA Toolkit (https://ncbi.github.io/sra-tools/). The FastQC tool (https://www.bioinformatics.babraham.ac.uk/projects/fastqc/) was used to inspect the quality of the sequence data. To clip adaptor sequences that may be present and to remove low-quality sequences, the Trimmomatic tool (http://www.usadellab.org/cms/?page=trimmomatic) was used with the following command-line specifications: “ILLUMINACLIP:TruSeq3-SE-2.fa:2:30:10 HEADCROP:11 TRAILING:20 SLIDINGWINDOW:4:20 MINLEN:15.” Reads were mapped to a custom reference genome. The custom reference genome was constructed by deleting all *SMN* gene annotations from human reference genome hg19 and then, to the modified hg19, adding the 215P15 clone sequence as a separate contig, as described previously.^44^ For reference mapping against the customized version of the human genome, the RNA sequencing (RNA-Seq) tool, supported in the CLCbio Genomics Workbench (version [v.]12) was used under default parameters. Mapped reads were visualized using Integrative Genomics Viewer (IGV) version 2.4.8 (http://software.broadinstitute.org/software/igv/).

### MEF isolation and culture

Mouse work was performed in the Biomedical Sciences Unit at the University of Oxford as authorized by the UK Home Office (Animal Scientific Procedures Act 1986). Taiwanese SMA mice were bred and maintained as described on The Jackson Laboratory website and as described previously.^36^^,^^63^ MEFs were isolated from strain FVB.Cg-*Smn1*^*tm1*^*Hung*Tg(SMN2)2Hung/J (Jackson Laboratory; 005058), crossed with strain FVB.129P2(B6)-*Smn1*^*tm1Hung*^/J (Jackson Laboratory; 031678), using a method described previously.^64^

After 2 additional days of culturing in MEF culture medium, the cells were plated for ASO transfection. For the MEF lines with sufficient cell counts, duplicate wells were plated (one for transfection with the 5′ UTR ASO and one for transfection with the NTC ASO). Single wells were plated for the MEF lines with fewer cells. The MEFs were transfected with ASO using RNAi MAX as described above. 3 days later, the MEFs were collected in RIPA buffer and subsequently assayed by immunoblotting.

*KO/D7;SMN2*- and *KO/F7*-immortalized MEFs^37^ shared by the Burghes lab were transfected with ASO and immunoblotted 2 days post-transfection.

### Statistics

Data were analyzed in Microsoft Excel and GraphPad Prism 8. In experiments with an untreated condition, all sample values are shown as a fold difference relative to the untreated samples. For these figures, error bars show standard error of the mean (SEM) of the fold differences. One-way ANOVA with Dunnett’s multiple comparisons test was used to determine statistical significance.

In experiments without an untreated condition, all samples were expressed as a fold difference relative to the NTC samples. For these figures, error bars show propagated error. In short, when normalizing the protein of interest to the loading control, the error associated with each signal intensity value was divided by the mean signal intensity value, and this fraction was squared. The values for the two proteins were summed, and then the square root was calculated. The resulting error value was used in a second, identical round of error computation in order to propagate it through the fold-difference calculation. Statistical significance was determined by t test between the normalized signal intensity values for the two sample groups.

For the RNA stability assay, we used a linear mixed model with total *SMN* mRNA level as the outcome variable and hours post-actinomycin D treatment, group, and their interaction as independent variables. Sample was specified as a random intercept. The significant interaction statistic was tested using a likelihood ratio test between the full model and a reduced no-interaction model and reported using a chi-square statistic and p value. If significant, the slopes for each group were then compared to each other and were Bonferroni corrected for 3 comparisons.

### Data presentation

Graphs were made using GraphPad Prism 8. Some schematics were created using BioRender (BioRender.com). Figures were assembled in Microsoft PowerPoint and Adobe Illustrator.

## Supplemental information

Document S1. Tables S1–S8 and Figures S1–S5

Document S2. Article plus supplemental information
