# Supplementary material for: Targeting the 5′ untranslated region of SMN2 as a therapeutic strategy for spinal muscular atrophy
Source: Mol Ther Nucleic Acids. 2021 Jan 5;23:731–42. doi: 10.1016/j.omtn.2020.12.027 (PMC7851419; doi:10.1016/j.omtn.2020.12.027)
Supplement: Document S1. Tables S1–S8 and Figures S1–S5 [file mmc1.pdf]

OMTN, Volume *23*

Supplemental Information

Targeting the 5′ untranslated region of *SMN2* as a therapeutic strategy for spinal muscular atrophy

Audrey M. Winkelsas, Christopher Grunseich, George G. Harmison, Katarzyna Chwalenia, Carlo Rinaldi, Suzan M. Hammond, Kory Johnson, Melissa Bowerman, Sukrat Arya, Kevin Talbot, Matthew J. Wood, and Kenneth H. Fischbeck

Table S1. Properties of ASOs used in experiments.

The first 7 ASOs listed were fully modified with 2’-O-methyl (2’OMe) bases and phosphorothioate (PS) linkages. The non-targeting control (NTC) ASO uses the “Standard Control” sequence which was described by Gene Tools, LLC as having no RNA target and minimal biological activity. The middle 3 ASOs were fully modified with 2’-O-methoxyethyl (2’-MOE) bases and phosphorothioate (PS) linkages. The final 2 ASOs were in the phosphorodiamidate morpholino oligomer (PMO) chemistry.

Sequence name	ASO sequence (5' -> 3')	Chemistry	% GC
ASO #1	GUUAUCGCCCUCCCACAUUUGUGG	2’-OMe	54.2
ASO #2	UGGUUAUCGCCCUCCCACAUUUGU	2’-OMe	50.0
ASO #3	AGUGGUUAUCGCCCUCCCACAUUU	2’-OMe	50.0
ASO #4	CGAGUGGUUAUCGCCCUCCCACAU	2’-OMe	58.3
ASO #5	UACGAGUGGUUAUCGCCCUCCCAC	2’-OMe	58.3
ASO #6	UUCUGGGAGCGGAACAGUACGGUG	2’-OMe	58.3
NTC	CCUCUUACCUCAGUUACAAUUUAUA	2’-OMe	32.0
5’UTR ASO	GUUAUCGCCCUCCCACAUUUGUGG	2’-MOE	54.2
NTC	CCUCUUACCUCAGUUACAAUUUAUA	2’-MOE	32.0
Scrambled	GUGGUCGCAUUUCCUCGUUACCAC	2’-MOE	54.2
5’UTR pPMO	TGGTTATCGCCCTCCCACATTTGTG	PMO	52.0
DM1 pPMO[63]	CAGCAGCAGCAGCAGCAGCAG	PMO	66.7%

Table S2. SMA iPSC lines used for motor neuron differentiation.

iPSC line name	Gender	Age at biopsy	Reprogramming method	Genotype	Disease
SMA A4	F	109 days	STEMCCA Lentivirus Reprogramming	Homozygous *SMN1* deletion, 2 copies *SMN2*	SMA Type 1
RS 1.2	M	74 days	STEMCCA Lentivirus Reprogramming		Age-matched control
IPS-OXSMA-03 (Clone 03 03)	M	49 years	CytoTune Sendai Reprogramming	Homozygous *SMN1* deletion, 3 copies *SMN2*	SMA Type 2
IPS-OXSMA-02 (Clone 02 04)	M	30 years	CytoTune Sendai Reprogramming	Homozygous *SMN1* deletion, 4 copies *SMN2*	SMA Type 3
OX1 (Clone 841-03-01)	M	36 years	CytoTune Sendai Reprogramming		Age-matched control

Table S3. Primers for gene expression analysis by qRT-PCR.

RNA	Primer name	Primer sequences (5’ -> 3’)	Chemistry	T_a (ºC)
FL-SMN	FL-SMN qF	GCTTTGGGAAGTATGTTAATTTCA	Sybr Green	60
	FL-SMN qR	CTATGCCAGCATTTCTCCTTAATT		
SMNΔ7	SMNΔ7 qF	CCACCACCCCACTTACTATCA	Sybr Green	60
	SMNΔ7 qR	GCTCTATGCCAGCATTTCCATA		
Total SMN	Total SMN qF	GCGATGATTCTGACATTTGG	Sybr Green	60
	Total SMN qR	GGAAGCTGCAGTATTCTTCT		
GAPDH	GAPDH qF	CTCAACGACCACTTTGTCAAGCTC	Sybr Green	60
	GAPDH qR	TCTTACTCCTTGGAGGCCATGT		

Table S4. Primers for *SMN2* exon 7 inclusion RT-PCR.

Primer name	Primer sequences (5’ -> 3’)	T_a (ºC)
SMN PAGE Fwd	AGGTCTAAAATTCAATGGCCCA	60
SMN PAGE Rev	GTGTCATTTAGTGCTGCTCTATGC	

Table S5. Confirming Click-iT assay specificity to nascent RNA.

Before the 5-ethynyl uridine pulse, fibroblasts were treated with actinomycin D to prevent transcription and serve as an experimental control. Those samples have higher qRT-PCR C_t values (less amplification of *SMN* and *GAPDH*) than samples not treated with actinomycin D. Values shown are mean plus or minus standard deviation. n = 3.

	Average C_t *SMN*	Average C_t *GAPDH*
NTC ASO	27.41 ± 0.46	25.94 ± 0.08
5'UTR ASO	26.49 ± 0.81	25.40 ± 0.62
Untreated	27.20 ± 0.73	25.99 ± 0.61
Actinomycin D	34.04 ± 0.60	29.99 ± 0.39
Carrier	25.47 ± 0.05	25.92 ± 0.21

Table S6. Double-stranded DNA fragments used in plasmid construction.

All sequences purchased as gBlocks Gene Fragments or Ultramer DNA Oligos from IDT. Lower case letters indicate intronic sequence.

DNA name	Sequence (5' -> 3')
gBlocks: *SMN2* 5'UTR + eGFP + *HBB*	CCACAAATGTGGGAGGGCGATAACCACTCGTAGAAAGCGTGAGAAGTTACT<br>ACAAGCGGTCCTCCCGGCCACCGTACTGTTCCGCTCCCAGAAGCCCCGGGC<br>GGCGGAAGTCGTCACTCTTAAGAAGGGACGGGGCCCCACGCTGCGCACCCG<br>CGGGTTTGCTATGGTGAGCAAGGGCGAGGAGCTGTTCACCGGGGTGGTGCC<br>CATCCTGGTCGAGCTGGACGGCGACGTAAACGGCCACAAGTTCAGCGTGTC<br>CGGCGAGGGCGAGGGCGATGCCACCTACGGCAAGCTGACCCTGAAGTTCAT<br>CTGCACCACCGGCAAGCTGCCCGTGCCCTGGCCCACCCTCGTGACCACCCTG<br>ACCTACGGCGTGCAGTGCTTCAGCCGCTACCCCGACCACATGAAGCAGCAC<br>GACTTCTTCAAGTCCGCCATGCCCGAAGGCTACGTCCAGGAGCGCACCATC<br>TTCTTCAAGGACGACGGCAACTACAAGACCCGCGCCGAGGTGAAGTTCGAG<br>GGCGACACCCTGGTGAACCGCATCGAGCTGAAGGGCATCGACTTCAAGGAG<br>GACGGCAACATCCTGGGGCACAAGCTGGAGTACAACTACAACAGCCACAA<br>CGTCTATATCATGGCCGACAAGCAGAAGAACGGCATCAAGGTGAACTTCAA<br>GATCCGCCACAACATCGAGGACGGCAGCGTGCAGCTCGCCGACCACTACCA<br>GCAGAACACCCCCATCGGCGACGGCCCCGTGCTGCTGCCCGACAACCACTA<br>CCTGAGCACCCAGTCCGCCCTGAGCAAAGACCCCAACGAGAAGCGCGATCA<br>CATGGTCCTGCTGGAGTTCGTGACCGCCGCCGGGATCACTCTCGGCATGGA<br>CGAGCTGTACAAGCTGCTGGTGGTCTACCCTTGGACCCAGAGGTTCTTTGAG<br>TCCTTTGGGGATCTGTCCACTCCTGATGCTGTTATGGGCAACCCTAAGGTGA<br>AGGCTCATGGCAAGAAAGTGCTCGGTGCCTTTAGTGATGGCCTGGCTCACC<br>TGGACAACCTCAAGGGCACCTTTGCCACACTGAGTGAGCTGCACTGTGACA<br>AGCTGCACGTGGATCCTGAGAACTTCAGGgtgagtctatgggacgcttgatgttttctttccccttctt<br>ttctatggttaagttcatgtcataggaaggggataagtaacagggtacagtttagaatgggaaacagacgaatgattgcatcagt<br>gtggaagtctcaggatcgttttagtttcttttatttgctgttcataacaattgttttcttttgtttaattcttgctttcttttttttcttctccgca<br>atttttactattatacttaatgccttaacattgtgtataacaaaaggaaatatctctgagatacattaagtaacttaaaaaaaaactttta<br>cacagtctgcctagtacattactatttggaatatatgtgtgcttatttgcatattcataatctccctactttattttcttttatttttaattgat<br>acataatcattatacatatttatgggttaaagtgtaatgttttaatatgtgtacacatattgaccaaatcagggtaattttgcatttgtaa<br>ttttaaaaaatgctttcttcttttaatatacttttttgtttatcttatttctaatactttccctaatctctttctttcagggcaataatgatacaat<br>gtatcatgcctctttgcaccattctaaagaataacagtgataatttctgggttaaggcaatagcaatatctctgcatataaatatttct<br>gcatataaattgtaactgatgtaagaggtttcatattgctaatagcagctacaatccagctaccattctgcttttattttatggttggg<br>ataaggctggattattctgagtccaagctaggcccttttgctaatcatgttcatacctcttatcttcctcccacagCTCCTGG<br>GCAACGTGCTGGTCTGTGTGCTGGCCCATCACTTTGGCAAAGAATTCACCCC<br>ACCAGTGCAGGCTGCCTATCAGAAAGTGGTGGCTGGTGTGGCTAATGCCCT<br>GGCCCACAAGTATCACTAAGCTCGCTTTCTTGCTGTCCAATTTCTATTAAAG<br>GTTCCTTTGTTCCCTAAGTCCAACTACTAAACTGGGGGATATTATGAAGGGC<br>CTTGAGCATCTGGATTCTGCC
5'UTR Frame Shift mutation	GTCAGATCCGCTAGGGATCCCCACAAATGTGGGAGGGCGATAACCACTCGT<br>ACCGAAAGCGTGAGAAGTTACTACAAGCGGTCCTCCCGGCCACCGTACTGT<br>TCCGCTCCCAGAAGCCCCGGGCGGCGGAAGTCGTCACTCTTAAGAAGGGAC<br>GGGGCCCCACGCTGCGCACCCGCGGGTTTGCTATGGTGAGCAAGGGCGAGG<br>AGCTGTTCACCGGGGTGGTGCCCATCCTGGTCGAGCTGGACGGCGACGTAA<br>ACGGCCACAAGTTCAGCGTGTCCGGCGAGGGCGAGGGCGATGCCACCTACG<br>GCAAGCTGACCCTGAAGTTCATCTGCACCACCGGCAAGCTGCCCGTGCCCT<br>GGCCCACCCTCGTGACCACCCTGACCTACGGCGTGCAGTGCTTCAGCCGCTA<br>CCCCGACCACATGAAGCAGCACGACTTCTTCAAGTCC
No mCherry	GACTGCAGCCTCAGGAGATCTGAATTCTCCAGGCGATCTGACGG

Table S7. Primers for PCRs for cloning.

PCR	Primer name	Primer sequence (5' -> 3')	T_a (ºC)
mCherry backbone	BglII-mCherry	ACTGCAGCCTCAGGAGATCTTTACTC GTCCATGCCGCCGG	70
	mCherry-EcoRI	CAGATCGCCTGGAGAATTCATGGTG AGCAAGGGCGAGGAG	
Wild-type	BamHI WT	CAGATCCGCTAGGGATCCCCACAAA TGTGGGAGGGCG	62
	β Globin Rev	AAGCTTATCGATGCGGCCGCGGCAG AATCCAGATGCTCAAGG	
ATG -> ACG	BamHI Mut	CAGATCCGCTAGGGATCCCCACAAA CGTGGGAGGGCG	62
	β Globin Rev	AAGCTTATCGATGCGGCCGCGGCAG AATCCAGATGCTCAAGG	
Optimized uORF	BamHI Opt	CAGATCCGCTAGGGATCCCCACAAA TGGGGAGGGCGATAACCACTCGTTA GAAAGCGTGA	62
	β Globin Rev	AAGCTTATCGATGCGGCCGCGGCAG AATCCAGATGCTCAAGG	
Frame Shift	eGFP internal	AGCACGACTTCTTCAAGTCCGCCATG CC	62
	β Globin Rev	AAGCTTATCGATGCGGCCGCGGCAG AATCCAGATGCTCAAGG	

Table S8. Primers for reporter construct sequence verification.

Primer name	Primer sequence (5' -> 3')
5'UTR Rev	CATAGCAAACCCGCGGGTGC
SMN 5'UTR	AGAAGCCCCGGGCGGCGGAA
eGFP internal	AGCACGACTTCTTCAAGTCCGCCATGCC
β Glob Fwd ORF	CGGCATGGACGAGCTGTACAAGCTGCTGGTGGTCTACC CTTGGAC
pBI CMV4 Rev	TGGAGATATCGTCGACAAGC
mCherry seq Fwd	AGTGCCACCTGACGTCGGCAG
mCherry seq Rev	ATGTAACGCGGAACTCCATATATG

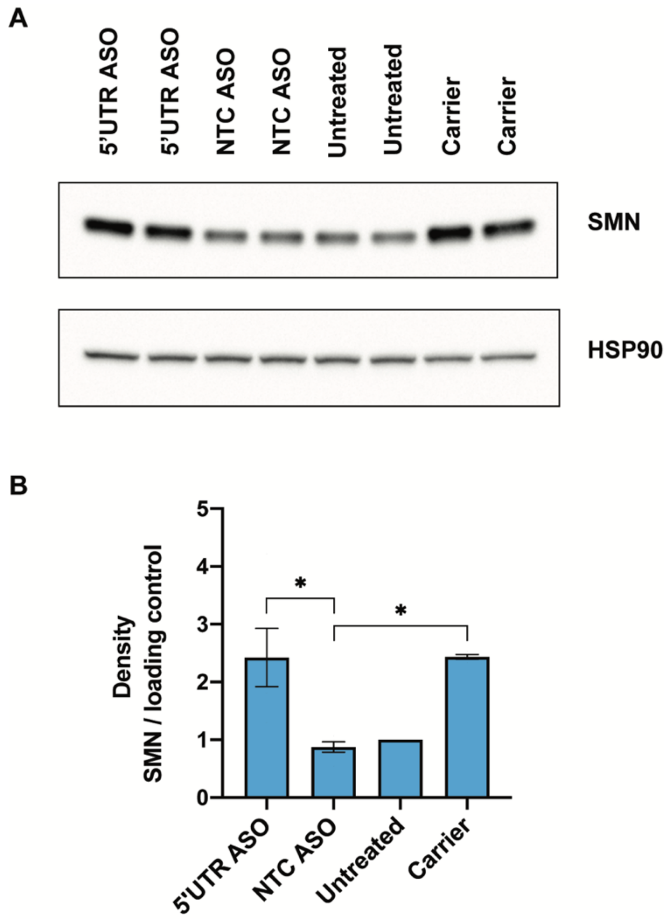

Figure S1. The 5'UTR ASO is effective in a second SMA fibroblast cell line.

A) Immunoblot (15 µg per lane) showing SMN protein levels in GM03813 fibroblasts treated with 600 nM 5'UTR ASO #1 or a non-targeting control (NTC) ASO. B) SMN protein levels normalized to a loading control (alpha tubulin or HSP90) and then calculated as a fold change relative to SMN levels in untreated SMA patient cells. Error bars show SEM. Statistical significance determined by one-way ANOVA followed by Dunnett's test in comparison to NTC ASO. n = 5, except n = 4 for carrier; * $p < 0.02$.

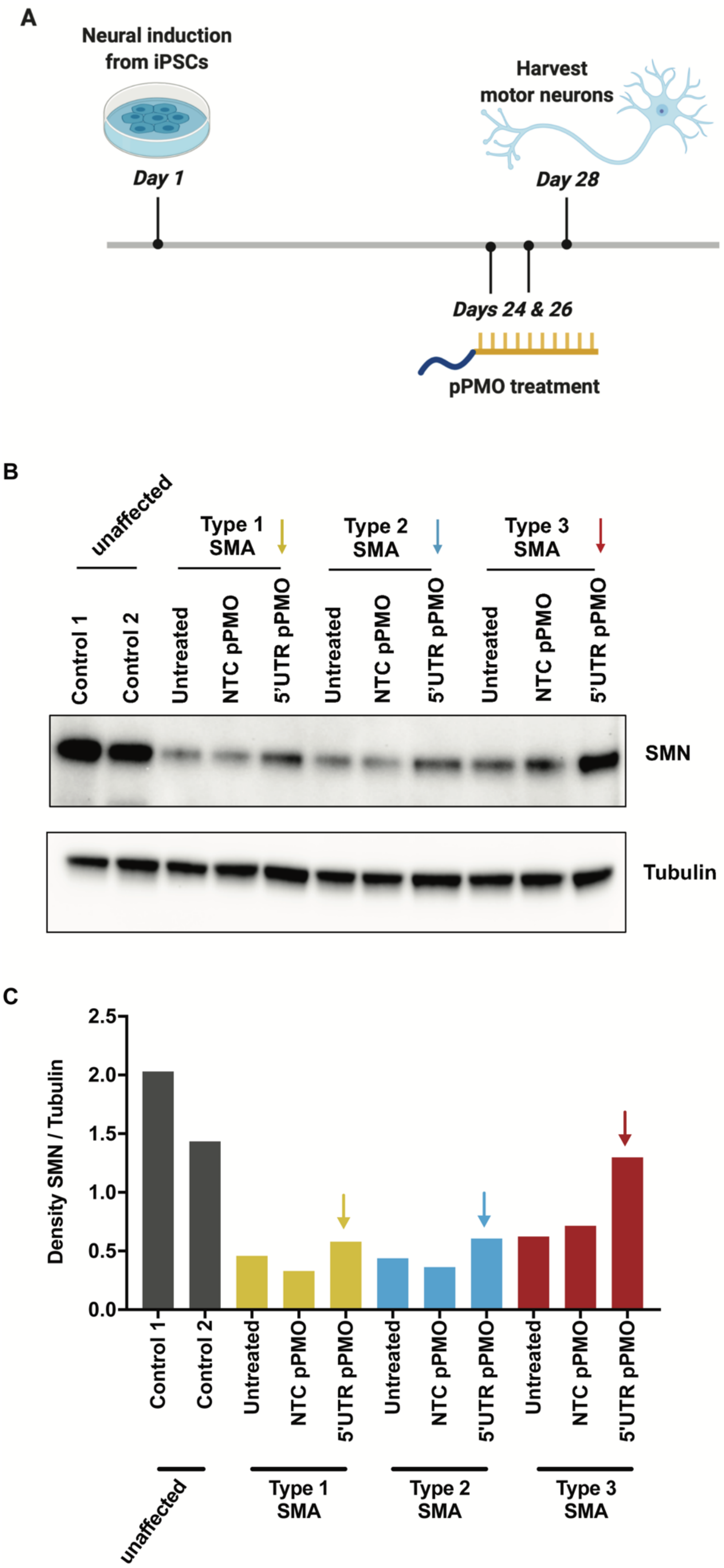
A
Neural induction from iPSCs
Day 1
Harvest motor neurons
Day 28
Days 24 & 26
pPMO treatment
B
unaffected
Type 1 SMA
Type 2 SMA
Type 3 SMA
Control 1
Control 2
Untreated
NTC pPMO
5'UTR pPMO
Untreated
NTC pPMO
5'UTR pPMO
Untreated
NTC pPMO
5'UTR pPMO
SMN
Tubulin
C
Density SMN / Tubulin
2.5
2.0
1.5
1.0
0.5
0.0
Control 1
Control 2
Untreated
NTC pPMO
5'UTR pPMO
Untreated
NTC pPMO
5'UTR pPMO
Untreated
NTC pPMO
5'UTR pPMO
unaffected
Type 1 SMA
Type 2 SMA
Type 3 SMA

Figure S2. The 5'UTR pPMO increases SMN protein levels in iPSC-derived motor neurons. A) A schematic drawing illustrating timing of treatment and protein extraction in the motor neuron differentiation process. In total, cells were exposed to pPMO for 4 days. B) Immunoblot (4-12% Bis-Tris gel) of motor neuron-like cells (MNs) chemically differentiated from iPSCs derived from control, Type 1 SMA, Type 2 SMA, or Type 3 SMA individuals. MNs were treated with 0.5 μM 5'UTR pPMO or 1 μM NTC pPMO. The NTC pPMO, which was developed for myotonic dystrophy (DM1),[63] targets the CUG expansion in *DMPK* and we did not expect it to change *SMN2* expression. C) Densitometry from immunoblot in panel B. SMN protein levels were normalized to alpha tubulin. n = 1 per cell line.

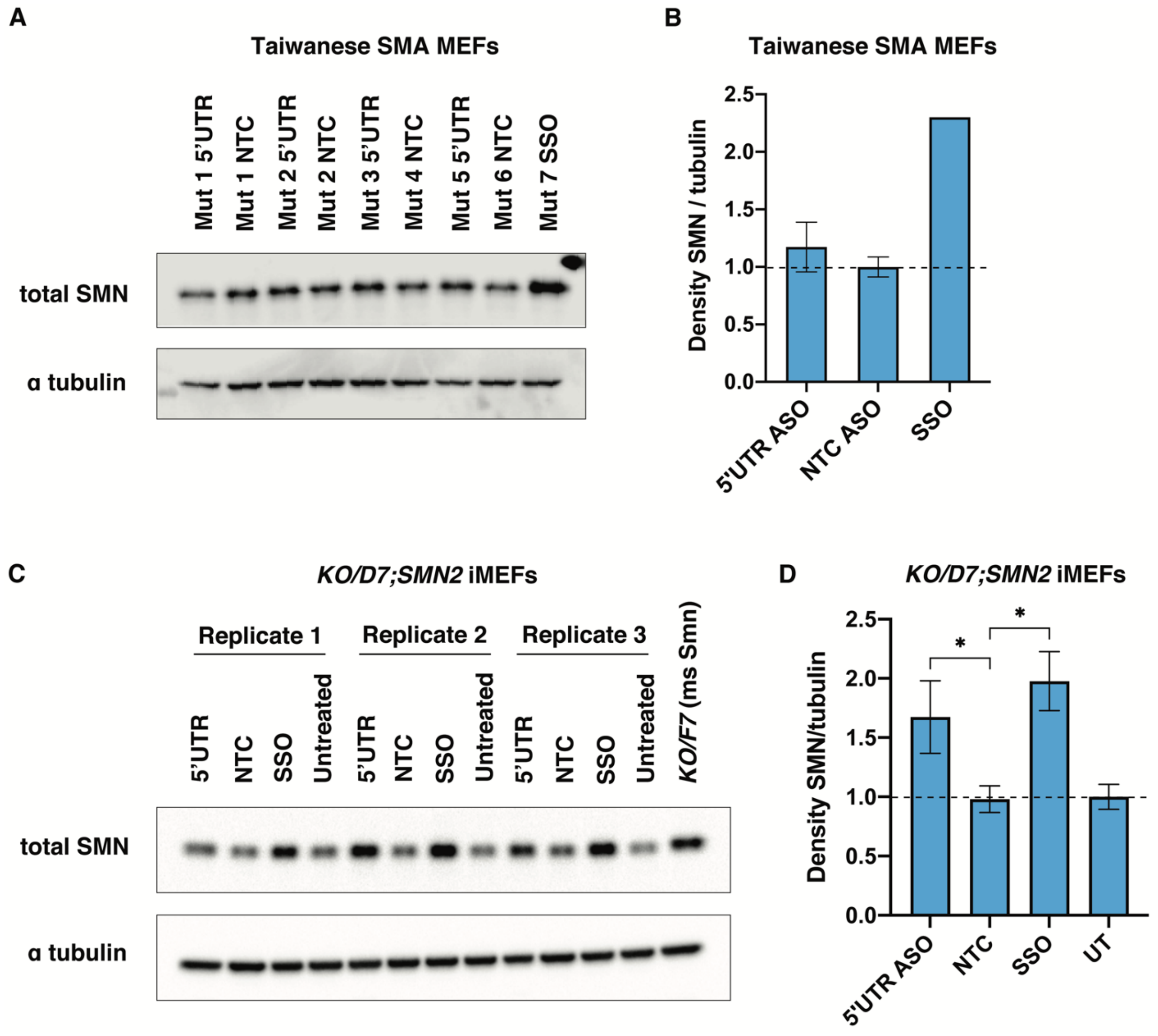

Figure S3. Effect of the 5'UTR ASO in cultured mouse cells.

A) Immunoblot (20 µg per lane) showing SMN protein levels from Taiwanese mouse embryonic fibroblasts treated with 150 nM 2'-MOE ASOs. The SSO (targeting the ISSN1 sequence) was used as a transfection positive control and was in the 2'OMe chemistry. Mut = mutant (SMA genotype). The number in the sample name indicates the embryo from which cells were isolated and grown. B) Densitometry from immunoblot in panel A. SMN protein levels were normalized to alpha tubulin and then calculated as a fold change relative to SMN levels in cells treated with the NTC ASO. Error bars

show propagated error. C) Immunoblot (15 µg per lane) showing SMN protein levels from *KO/D7;SMN2* mouse embryonic fibroblasts[37] treated with 300 nM 2'-MOE ASOs. *KO/F7* has one copy of the mouse Smn gene. D) Densitometry from immunoblot in panel C. SMN protein levels were normalized to alpha tubulin and then calculated as a fold change relative to SMN levels in untreated cells. Error bars show propagated error. * $p < 0.05$.

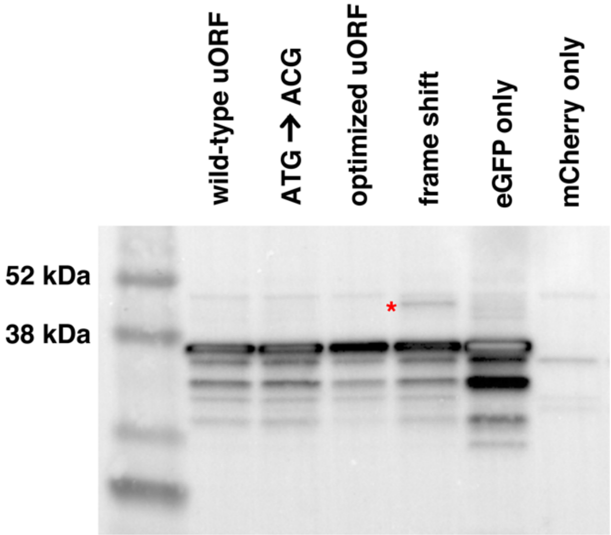

Figure S4. Translation that begins at the upstream start codon is rare (or slow).

The wild-type eGFP fusion protein is 39 kDa. The protein encoded by the frame shift reporter has 53 additional amino acids (encoded by the 5'UTR) and is 45 kDa. This protein is indicated by the red asterisk. The signal from the higher molecular weight protein is visible only after the signal from the canonical protein is saturated.

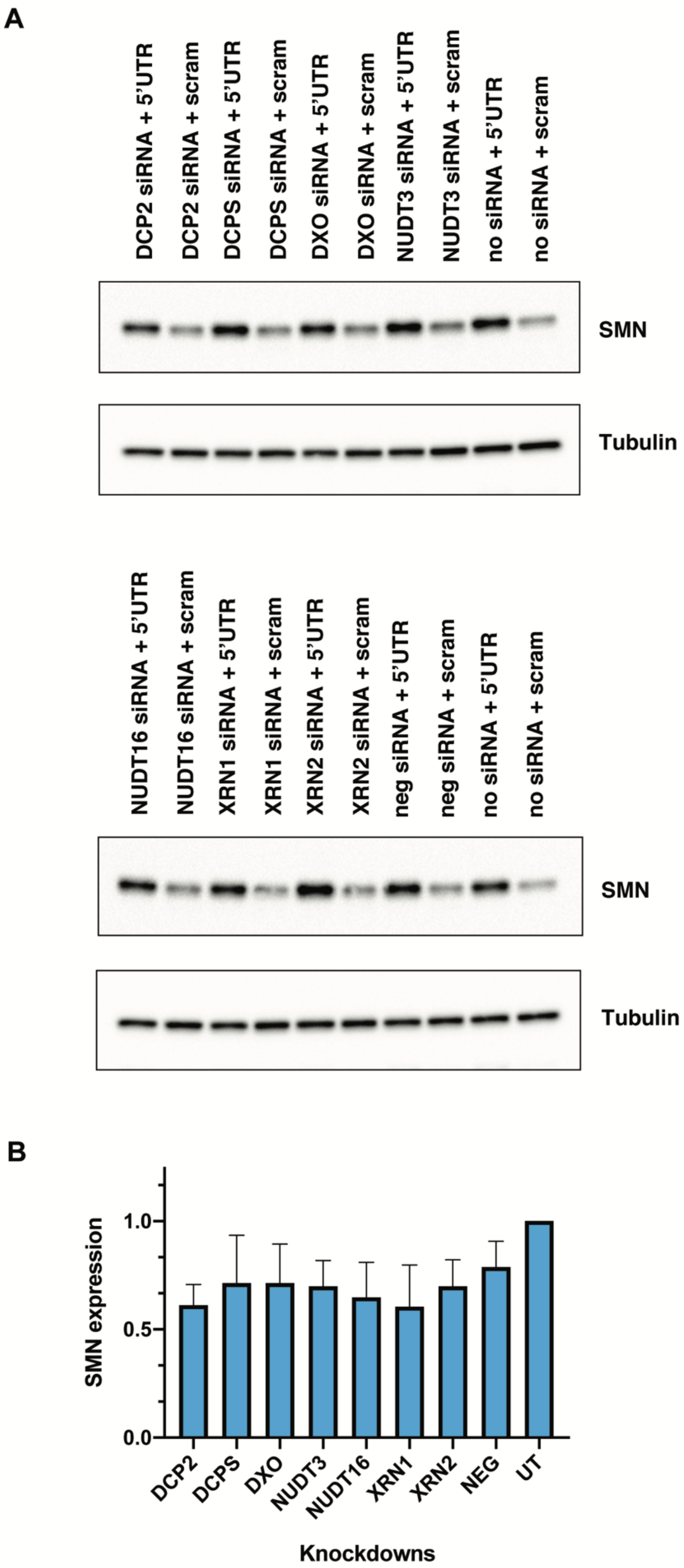
A
DCP2 siRNA + 5'UTR
DCP2 siRNA + scram
DCPS siRNA + 5'UTR
DCPS siRNA + scram
DXO siRNA + 5'UTR
DXO siRNA + scram
NUDT3 siRNA + 5'UTR
NUDT3 siRNA + scram
no siRNA + 5'UTR
no siRNA + scram
SMN
Tubulin
NUDT16 siRNA + 5'UTR
NUDT16 siRNA + scram
XRN1 siRNA + 5'UTR
XRN1 siRNA + scram
XRN2 siRNA + 5'UTR
XRN2 siRNA + scram
neg siRNA + 5'UTR
neg siRNA + scram
no siRNA + 5'UTR
no siRNA + scram
SMN
Tubulin
B
SMN expression
1.0
0.5
0.0
DCP2
DCPS
DXO
NUDT3
NUDT16
XRN1
XRN2
NEG
UT
Knockdowns

Figure S5. Knockdown of decapping factors and exoribonucleases does not abrogate 5'UTR ASO activity. Enzymes involved in RNA decay were knocked down with 100 nM siRNA in GM0232 (SMA) fibroblasts. The next day, the cells were transfected with 300 nM 5'UTR 2'-MOE or scrambled 2'-MOE. A) Immunoblots (15 µg per lane) showing SMN protein levels in SMN-deficient fibroblasts treated with siRNA and ASO combinations indicated. B) SMN protein levels were normalized to alpha tubulin and then calculated as a fold change relative to normalized SMN levels in cells treated with ASO but not siRNA. UT = no siRNA treatment. Error bars show SEM. There was no statistical significance between conditions, as determined by one-way ANOVA followed by Dunnett's test in comparison to NTC. n = 3/4.
