# Supplementary material for: Targeting the 5′ untranslated region of SMN2 as a therapeutic strategy for spinal muscular atrophy
Source: Mol Ther Nucleic Acids. 2021 Jan 5;23:731–42. doi: 10.1016/j.omtn.2020.12.027 (PMC7851419; doi:10.1016/j.omtn.2020.12.027)
Supplement: Document S2. Article plus supplemental information [file mmc2.pdf]

Molecular Therapy
Nucleic Acids
Original Article

Targeting the 5′ untranslated region of *SMN2* as a therapeutic strategy for spinal muscular atrophy

Audrey M. Winkelsas,[1,2] Christopher Grunseich,[1] George G. Harmison,[1] Katarzyna Chwalenia,[3] Carlo Rinaldi,[3] Suzan M. Hammond,[3] Kory Johnson,[1] Melissa Bowerman,[4,6] Sukrat Arya,[5,7] Kevin Talbot,[5] Matthew J. Wood,[3] and Kenneth H. Fischbeck[1]

[1]Neurogenetics Branch, National Institute of Neurological Disorders and Stroke, National Institutes of Health, Bethesda, MD 20892, USA; [2]Nuffield Department of Medicine, University of Oxford, Oxford OX3 7BN, UK; [3]Department of Paediatrics, University of Oxford, Oxford OX1 3QX, UK; [4]Department of Physiology, Anatomy and Genetics, University of Oxford, Oxford OX1 3QX, UK; [5]Nuffield Department of Clinical Neurosciences, University of Oxford, Oxford OX3 9DU, UK

Spinal muscular atrophy (SMA) is a neuromuscular disorder caused by mutations in the survival motor neuron 1 (*SMN1*) gene. All patients have at least one copy of a paralog, *SMN2*, but a C-to-T transition in this gene results in exon 7 skipping in a majority of transcripts. Approved treatment for SMA involves promoting exon 7 inclusion in the *SMN2* transcript or increasing the amount of full-length SMN by gene replacement with a viral vector. Increasing the pool of *SMN2* transcripts and increasing their translational efficiency can be used to enhance splice correction. We sought to determine whether the 5′ untranslated region (5′ UTR) of *SMN2* contains a repressive feature that can be targeted to increase SMN levels. We found that antisense oligonucleotides (ASOs) complementary to the 5′ end of *SMN2* increase SMN mRNA and protein levels and that this effect is due to inhibition of *SMN2* mRNA decay. Moreover, use of the 5′ UTR ASO in combination with a splice-switching oligonucleotide (SSO) increases SMN levels above those attained with the SSO alone. Our results add to the current understanding of SMN regulation and point toward a new therapeutic target for SMA.

INTRODUCTION

Spinal muscular atrophy (SMA) is an autosomal recessive neuromuscular disorder caused by loss-of-function mutations in the survival motor neuron 1 (*SMN1*) gene.[1] Although *SMN1* is a ubiquitously expressed gene, SMA is primarily a disease of lower motor neurons. Denervation results in symmetrical muscle weakness, often within weeks or months of birth.[2] *SMN1* encodes the SMN protein, which has a well-characterized function in small nuclear ribonucleoprotein (snRNP) assembly.[3,4] Other cellular processes where SMN is likely involved include axonal mRNA transport and local translation and endocytosis, which may account for the motor neuron vulnerability in SMA.[5–8]

A complete absence of SMN protein results in embryonic lethality.[9] All SMA patients have at least one copy[10,11] of an *SMN1* gene paralog, *SMN2*, which arose from a duplication of the SMN locus on chromosome 5.[1] *SMN2* does not fully compensate for the loss of *SMN1*; due to a C-to-T transition that results in exon 7 skipping in a majority of transcripts, only 10 percent to 20 percent of *SMN2* mRNAs encode the fully functional SMN protein.[12–14] Clinically, disease severity therefore correlates with *SMN2* copy number and full-length *SMN2* transcript level.[15,16]

Increasing the level of SMN, via targeting *SMN2* or via gene therapy,[17] has been a primary therapeutic strategy for SMA. Nusinersen is an antisense oligonucleotide (ASO) that increases the proportion of *SMN2* transcripts containing exon 7.[18–20] Another SMN splice modifier, risdiplam, is a small molecule that has the advantage of being orally bioavailable.[21,22] Targeting splicing as a means of increasing SMN levels has a ceiling effect determined by the abundance of *SMN2* transcripts in cells. Increasing the total pool of *SMN2* transcripts and increasing the translational efficiency of these transcripts are two strategies to overcome the ceiling effect associated with the splice-switching strategy.

To identify a new target, we looked in the *SMN2* 5′ untranslated region (5′ UTR). It is known that regulatory motifs within 5′ UTRs influence gene expression by controlling transcript stability, translational efficiency, and subcellular localization.[23,24] This occurs through

Received 22 July 2020; accepted 30 December 2020;
https://doi.org/10.1016/j.omtn.2020.12.027.

[6]Present address: School of Medicine, Keele University, Staffordshire ST5 5BG, UK
[7]Present address: Department of Physiology, Anatomy and Genetics, University of Oxford, Oxford OX1 3QX, UK
Correspondence: Kenneth H. Fischbeck, Neurogenetics Branch, National Institute of Neurological Disorders and Stroke, National Institutes of Health, Bethesda, MD 20892, USA.
E-mail: kf@ninds.nih.gov
Correspondence: Audrey M. Winkelsas, Neurogenetics Branch, National Institute of Neurological Disorders and Stroke, National Institutes of Health, Bethesda, MD 20892, USA.
E-mail: audrey.winkelsas@nih.gov

This is an open access article under the CC BY license (http://creativecommons.org/licenses/by/4.0/).

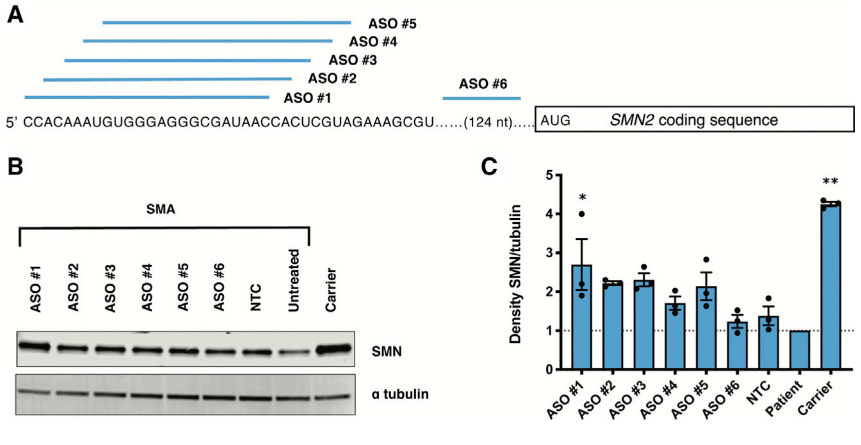

Figure 1. Targeting the 5′ end of *SMN2* with ASOs increases levels of SMN in fibroblasts

(A) A schematic depicting the tiling of 2′-OMe ASOs in 2 nt increments along the beginning of the *SMN2* 5′ UTR. (B) Immunoblot (15 μg per lane) showing SMN protein levels in SMN-deficient fibroblasts (GM00232) treated with 600 nM 5′ UTR ASOs or a nontargeting control (NTC) oligo, where indicated. (C) SMN protein levels normalized to alpha tubulin and then calculated as a fold change relative to SMN levels in untreated SMA patient cells (represented by the dotted line). SMN levels from carrier cells are provided for reference. Error bars show SEM. Statistical significance was determined by one-way ANOVA followed by Dunnett's test in comparison to NTC. n = 3; *p = 0.02; **p < 0.0001.

a dynamic interplay between *cis-acting* elements (i.e., the primary sequence and secondary structures of 5′ UTRs) and *trans-acting* factors (i.e., RNA-binding proteins and noncoding RNAs). We sought to determine whether the 5′ UTR of *SMN2* contains a repressive feature that limits its expression, of which targeting could increase SMN levels. We identified sequences in the 5′ UTR that can be targeted with an ASO, which through binding to the 5′-most (ASO #1) end of the *SMN2* transcript, increases SMN levels by stabilizing *SMN2* mRNA. We found that the 5′ UTR ASO used in combination with a splice-switching oligonucleotide (SSO) augments SMN above levels achieved with an SSO alone. Our results add to the current understanding of *SMN2* mRNA turnover and point toward a new therapeutic target for SMA that can be pursued as a combinatorial therapy.

RESULTS

Targeting the 5′ end of *SMN2* with ASOs increases SMN protein levels

The 5′ UTR of *SMN2*[25–27] contains a start codon 157 nucleotides upstream of the canonical *SMN* translation initiation codon. Nearly one-half of all human transcripts contains upstream open reading frames (uORFs), features that may attenuate translation of the main protein coding sequence.[28] Recently, it was shown that ASO binding at start codons in 5′ leader sequences can prevent translation initiation at uORFs and promote translation of primary ORFs.[29] In addition, ASOs have been used to increase translation of mRNAs containing other types of 5′ UTR inhibitory elements, such as G-quadruplexes or hairpin structures.[30,31]

We designed a series of overlapping 2′-*O*-methyl (2′-OMe) ASOs in 2-nucleotide increments across the 5′ region of the *SMN2* transcript, including the uORF associated with the putative start codon (Figure 1A). ASOs in which all bases contain the 2′-OMe modification operate via steric hindrance rather than RNase H-mediated RNA degradation and can therefore be used to increase gene expression. Table S1 shows the sequence of each ASO. Transfection of ASOs targeting the 5′ UTR in SMA patient-derived fibroblasts resulted in increased SMN protein levels compared to the level of SMN in untransfected patient cells or those treated with a nontargeting control (NTC) ASO (Figures 1B and 1C). Stepping 5′ to 3′ across the UTR, there is a downward trend in the ASO effect on SMN expression, indicating that the critical target region is close to the 5′ terminal cap. We decided to use ASO #1 in the experiments that follow since it demonstrated the largest biological effect, with an average 2.7-fold increase in SMN protein levels. We tested this ASO #1 in a second SMA cell line (Figure S1) to confirm that its effects are not cell-line specific. We found a similar trend using a 5′ UTR phosphorodiamidate morpholino oligomer (PMO) conjugated to a cell-penetrating peptide (pPMO) in motor neuron-like cells chemically differentiated from SMA patient-derived induced pluripotent stem cells (iPSCs; Table S2; Figure S2).[32,33]

We also tested an ASO with the same sequence as ASO #1 but with 2′-*O*-(2-methoxyethyl) (2′-MOE)-modified bases. 2′-MOE-modified ASOs are known to undergo less nonspecific protein binding and are among the most widely used in clinical trials.[34,35] We found that treating human SMA fibroblasts with the 5′ UTR 2′-MOE increases SMN protein levels 3.7-fold (Figures 2A and 2B). The increased efficacy observed in Figure 2 compared to Figure 1 is likely attributable to the shift from 2′-OMe to 2′-MOE chemistry.

Studies in fibroblasts derived from Taiwanese SMA mice, which are null for mouse *Smn* but contain a 115-kb human DNA sequence containing *SMN2*,[36] showed no effect of 5′ UTR ASO treatment (Figures S3A and S3B). However, the 5′ UTR ASO is effective in mouse embryonic fibroblasts (MEFs) containing the PAC 215P15-derived human *SMN2* transgene[37,38] (Figures S3C and S3D). This could be due to differences in the background genetics of the two mouse strains.

The SMN protein is part of a large protein complex where it associates with Gemin proteins. Previous investigation demonstrated that levels of Gemin6 and Gemin8, which are core components of the SMN complex, correlate with SMN expression levels.[39] After seeing the effect of the ASO on SMN levels, we sought to determine whether Gemin levels are also increased. If so, this would indicate functional correction of the SMN deficiency with ASO treatment. Indeed, by immunoblot, we found the increase in SMN to be accompanied by

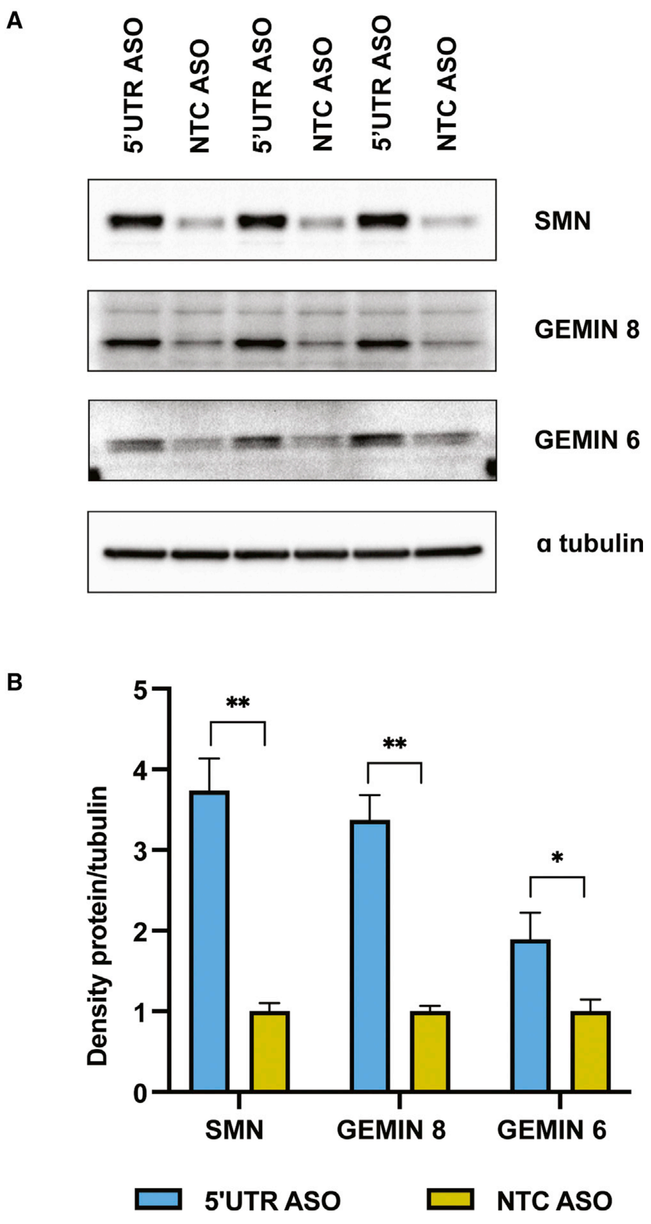

Figure 2. Levels of SMN complex members increase with 5′ UTR ASO treatment

(A) Immunoblot showing levels of SMN, Gemin6, and Gemin8 in SMA fibroblasts following transfection with 300 nM ASO. ASOs were fully modified with 2′-MOE bases and phosphorothioate (PS) linkages. 35 μg protein per sample was resolved due to difficulty in detecting Gemin6. (B) Levels of the proteins of interest were normalized to alpha tubulin, and this ratio was then averaged for the two sample groups (5′ UTR ASO and NTC ASO, in triplicate). The graph shows the level of each protein as a fold change relative to protein levels in cells transfected with the NTC ASO. Error bars show propagated error. Statistical significance was determined by t test between the normalized signal intensity values for the two sample groups. n = 3; $^{*}p < 0.02$; $^{**}p < 0.0002$.

1.9- and 3.4-fold increases in Gemin6 and Gemin8, respectively (Figures 2A and 2B).

The 5′ UTR ASO increases *SMN2* mRNA levels in fibroblasts by stabilizing transcripts

We next performed qRT-PCR to determine whether *SMN2* transcript levels increase following ASO treatment. Compared to untreated cells or cells treated with a NTC ASO, the 5′ UTR ASO increases total *SMN* mRNA levels in both SMA patient fibroblasts and carrier fibroblasts (Figures 3A and 3D). Total *SMN* mRNA levels were measured with primers spanning the exon 2a-2b junction and are thus irrespective of exon 7 inclusion.

Transcripts containing exon 7 are the most therapeutically relevant, as they encode the full-length SMN protein. This prompted us to check levels of the full-length and Δ7 *SMN* isoforms by RT-PCR and by qRT-PCR. To our surprise, the 5′ UTR ASO led to a shift toward a full-length transcript (with exon 7 inclusion) in patient fibroblasts (Figures 3B and 3C). In carrier fibroblasts from an unaffected individual with higher baseline SMN levels, the ASO increases steady-state mRNA without affecting the ratio of full-length to total transcripts (Figure 3D). Thus, we suspect that the increased level of total *SMN* mRNA is a direct effect of the 5′ UTR ASO, whereas increased exon 7 inclusion is more likely due to SMN feedback[40,41] (caused by an increased pool of snRNPs) in states of SMN deficiency rather than a primary mechanism of action of the ASO.

A higher steady-state level of mRNA could either be due to an increased rate of transcription or to a decreased rate of RNA decay. To distinguish between the two possibilities, we pulsed cells with a uridine analog, 5-ethynyl uridine (EU), to measure newly transcribed *SMN2*. Biotinylating the EU allowed us to isolate and quantify only those RNAs transcribed during the 1-h pulse via qRT-PCR. We confirmed assay specificity by blocking transcription with actinomycin D and observing an increase in cycle threshold (C_t) values of three to nine cycles for *GAPDH* and *SMN*, indicating decreased transcript levels (Table S5). As a positive control, we used SMA carrier fibroblasts that have one copy of *SMN1* and five copies of *SMN2* and thus should transcribe more *SMN* than our patient fibroblasts, which contain two copies of *SMN2* and no *SMN1*. With this RNA-labeling method, we saw no significant difference in nascent *SMN2* transcript levels among cells treated with the 5′ UTR ASO, cells treated with the NTC ASO, or untreated patient cells (Figure 4A). This indicates that the higher steady-state level of *SMN2* is not due to increased transcription but is due instead to slower RNA turnover. Indeed, by treating cells with actinomycin D and collecting RNA at different time points, we found that *SMN2* transcripts are significantly more stable in cells treated with the 5′ UTR ASO (Figure 4B).

The *SMN2* uORF is not readily translated and does not reduce SMN levels

When present, a uORF stop codon may be processed like a premature termination codon (PTC), subjecting the mRNA to nonsense-mediated decay. ASO-mediated inhibition of the uORF could thus explain

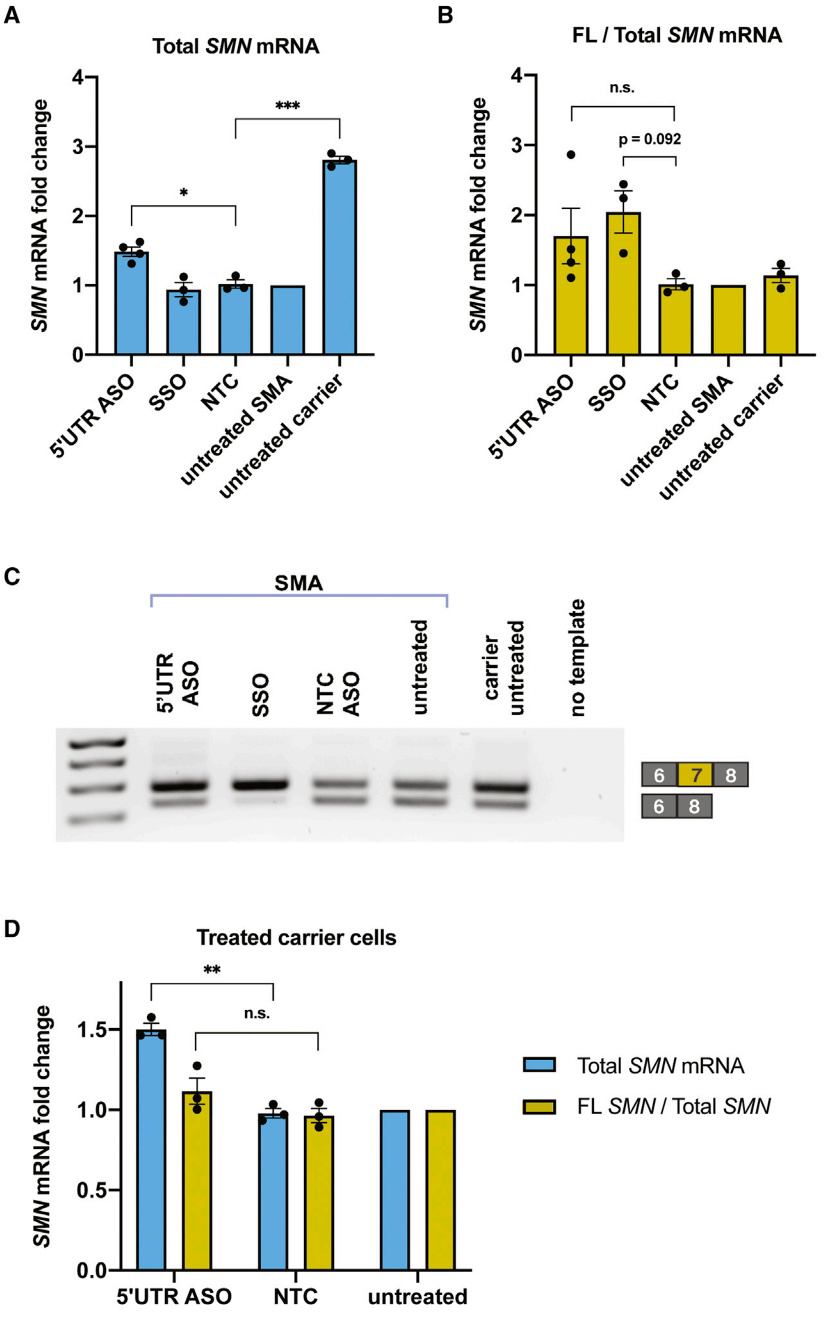

Figure 3. An ASO targeting the 5′ end of *SMN2* increases *SMN* mRNA levels

(A) SMA fibroblasts were transfected with 600 nM 2′-OMe 5′ UTR ASO, splice-switching oligonucleotide (SSO), or NTC ASO. qRT-PCR measured total *SMN* mRNA levels. Expression was normalized to *GAPDH* and calculated as a fold change relative to levels in untreated SMA cells. (B) qRT-PCR analysis of the ratio of full-length (FL) *SMN* to total *SMN* transcript levels, measured with primers overlapping exon 7 or primers spanning the exon 2a-2b junction, respectively. (C) RT-PCR to qualitatively visualize alternative splicing with 600 nM ASO treatment. The amplicon from the full-length isoform is 292 bp, whereas the amplicon from the Δ7 isoform is 238 bp. (D) As in (A) and (B), levels of total *SMN* mRNA or the ratio of full-length to total *SMN* mRNA were measured via qRT-PCR. Data in this panel are from ASO treatment in fibroblasts from a carrier of SMA (1 copy *SMN1*, 5 copies *SMN2*). Expression was normalized to *GAPDH* and calculated as a fold change relative to levels in untreated SMA cells. Error bars show SEM. Statistical significance was determined by one-way ANOVA followed by Dunnett's test in comparison to NTC. n = 3/4; *p < 0.005; **p < 0.001; ***p < 0.0001; n.s., not significant.

the increased *SMN2* transcript stability. To better understand the mechanism of action of the 5′ UTR ASO, we designed reporter constructs to study the effects of the uORF on SMN levels. In addition to a construct with enhanced green fluorescent protein (EGFP) under the control of the wild-type *SMN2* 5′ UTR, constructs were made with the following: (1) a mutation to remove the uORF start codon, (2) a mutation that strengthens the sequence context (Kozak sequence) surrounding the uORF start codon, and (3) a frameshift mutation that extends the uORF coding sequence and places it in-frame with GFP (Figure 5A). The latter reporter was designed in order to be able to observe uORF translation initiation, since the uORF peptide is too small to detect by standard techniques.

The expression of these reporters in HEK293T cells showed that removing the upstream start codon (mutating ATG to ACG) does not increase EGFP levels (Figures 5B and 5C), suggesting that the uORF does not have a significant influence on gene expression. There is, however, a decreased EGFP signal when a guanine is present in the +4 position ("uORF optimized" reporter). We can infer from this that the nonoptimized, native uORF is not functional. Finally, the larger protein encoded by the "frame shift" reporter was only detectable at very low levels, indicating that ribosomes do not often engage the uORF (Figure S4).

To rule out a lack of uORF effect being due to an artifact of the reporter system (e.g., use of a non-endogenous transcription start site), we aligned publicly available ribosome profiling data[42,43] to the *SMN2* locus (Figure 5D). Sequencing reads corresponding to *SMN1* and/or *SMN2* cannot be accurately mapped to standard reference genomes because multi-mapping reads are discarded or randomly distributed between the paralogs. Thus, we used a custom reference genome in which only the *SMN2* sequence is present.[44] In the ribosome profiling data, the absence of ribosome-protected fragments mapping to the *SMN2* uORF was in line with our reporter assay findings and supports the

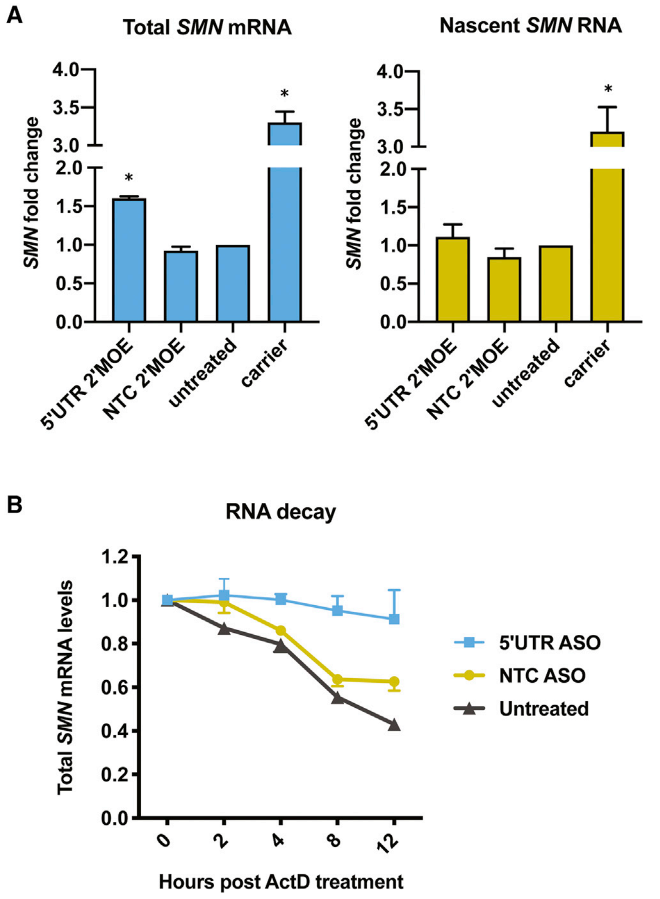

Figure 4. The ASO targeting the 5′ end of *SMN2* increases the level of steady-state *SMN* mRNA by decreasing its turnover

(A) SMA fibroblasts were transfected with 150 nM 2′-MOE 5′ UTR ASO or NTC ASO and pulsed with EU. qRT-PCR measured steady-state (total) *SMN* mRNA levels or nascent (biotinylated) *SMN* RNA levels. Expression was normalized to *GAPDH* and compared to levels in untreated SMA cells. Statistical significance was determined by one-way ANOVA followed by Dunnett's test in comparison to the NTC sample in its group. n = 3; *p ≤ 0.007. (B) 48 h post-transfection with 600 nM 2′-OMe ASOs, SMA fibroblasts were treated with actinomycin D (ActD) and collected in TRIzol at the specified time points. qRT-PCR measured total SMN mRNA. n = 3. Statistical analysis was performed using a linear mixed model as described in Materials and methods. The interaction between group and time was significant ($\chi^2(2) = 29.2$, p value < 0.001). The pairwise differences in slope are as follows: 5′ UTR ASO – NTC ASO = 0.028 (standard error = 0.006, p < 0.001); 5′ UTR ASO – untreated = 0.039 (standard error = 0.006, p < 0.001); NTC ASO – untreated = 0.011 (standard error = 0.006, not significant).

conclusion that the uORF in *SMN2* is not a meaningful regulator of *SMN2* expression in fibroblasts or in HEK293T cells.

A combinatorial therapeutic approach further increases levels of the SMN protein

Novel strategies to complement splice modulation of *SMN2* may be especially useful for those with SMA who have low *SMN2* copy numbers. The SSO and the 5′ UTR ASO were designed to target distinct RNA processes, leading us to investigate whether a combination of these two ASOs overcomes the ceiling effect associated with the SSO. We tested the 5′ UTR ASO and a SSO that targets the ISSN1 sequence in *SMN2*[20] separately and jointly. We found that concurrent use of the two ASOs in SMA patient fibroblasts increases SMN protein levels significantly more than use of the SSO alone (Figures 6A and 6B). We speculate that the combined treatment is not significantly different from the 5′ UTR ASO alone because of the shift toward the full-length transcript seen with 5′ UTR ASO treatment (depicted in Figure 3C).

DISCUSSION

Previously, histone deacetylase (HDAC) inhibitors were shown to increase *SMN2* levels.[45–47] HDAC inhibitors are not specific to the *SMN2* gene, however, and transcriptionally activate a broad array of genes. Some of these nonspecific changes in gene expression may benefit the SMA phenotype, but other changes may be harmful.[48,49] Thus, a method for increasing SMN production in a more specific way that complements the splice-switching approach is therapeutically desirable. Here, we show that an ASO, in three different chemistries, with sequence complementary to the 5′ UTR of *SMN2*, increases SMN mRNA and protein levels in human fibroblasts and motor neuron-like cells. Based on our experiments in MEFs, future preclinical studies should use the Burghes and colleagues'[50] SMNdelta7 SMA mouse model.

In addition to the SMN protein, we show that levels of at least two SMN-associated proteins (Gemin6 and Gemin8) increase with ASO treatment. This is likely because as SMN levels increase, there are more SMN complexes to which Gemin6 and Gemin8 can bind, and this confers stability. Details about the stoichiometry of proteins in the SMN complex are unknown. The 5′ UTR ASO may thus be useful in future experiments to study how proteins such as Gemin6 and Gemin8 are incorporated into the SMN complex, as well as in studying other pathways in which the SMN protein is involved more generally.

Our initial hypothesis was that by blocking translation of the uORF, the 5′ UTR ASO promotes translation of the primary ORF. However, with multiple techniques, we found that the *SMN2* uORF is not normally translated. This may be due to the short distance between the 5′ cap and the uORF start codon (7 nucleotides) or to the weak sequence context surrounding the start codon (T at the +4 position).

Instead, the 5′ UTR ASO stabilizes *SMN2* mRNA. Although it has been established that the SMN protein is degraded by the E3 ubiquitin ligase mind bomb 1 and the proteasome, the process through which *SMN* transcripts are degraded is less clear.[51] A high-throughput screen identified a quinazoline compound that inhibits the mRNA decapping enzyme DcpS and increases *SMN2* promoter activity in cell-based assays.[52] Follow-up studies found that this small molecule increases survival and motor function in SMA mice.[53–56] Whereas it is known that DcpS hydrolyzes cap structures from mRNA fragments that are generated by extensive 3′ to 5′ exonuclease decay, the specific mechanism through which the quinazoline compound increases *SMN2* expression is unknown.

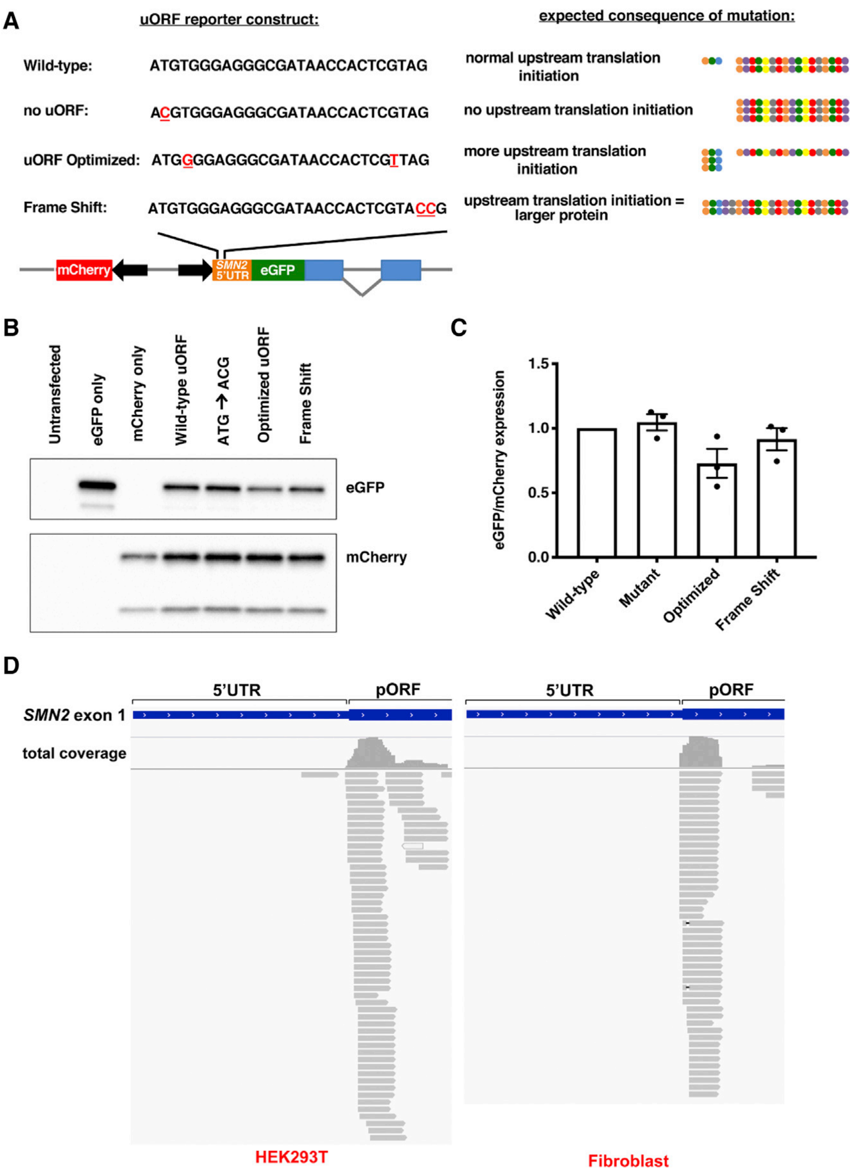

Figure 5. The *SMN2* uORF is not readily translated and does not reduce expression of the primary ORF (pORF)

(A) A schematic detailing reporter construct designs, with mutations underlined in red. Expected sizes and relative expression levels of protein products are indicated on the right. The uORF-encoded peptide is represented as the short string (3 circles), whereas the pORF-encoded peptide is represented as the longer string (17 circles). The protein encoded by the frameshift reporter (when translation initiates at the upstream start codon and continues through the 5′ UTR and the pORF) is represented as the longest, continuous string. (B) HEK293Ts were transfected with plasmids and expression levels determined by western blot. 7.5 μg protein was resolved per lane. (C) EGFP protein levels were normalized to mCherry levels and then normalized to expression from cells transfected with the wild-type plasmid. Error bars represent SEM. (D) Ribosome profiling data (HEK293T = GEO: GSM3566399; fibroblast = GEO: GSM1047585) aligned to the *SMN2* locus and visualized using IGV. Gray lines represent individual sequencing reads, with arrows indicating read direction. Total coverage at a particular locus is indicated above reads.

The idea that the 5′ UTR ASO operates via a mechanism related to decapping is compelling since the ASO is complementary to the *SMN2* sequence immediately adjacent to the 5′ cap. However, we did not find a significant difference in the ASO's ability to upregulate SMN levels in cells in which decapping factors were knocked down (Figure S5). We cannot rule out this mechanism of action entirely since it is possible that with the knockdown of individual enzymes, there is compensation by other RNA decay machinery.

We found that with the increase in *SMN* mRNA levels, there is a trend toward an increase in the ratio of full-length to exon 7-excluded transcripts in patient fibroblasts. Other compounds that increase levels of SMN have been shown to increase exon 7 inclusion, including an HDAC inhibitor and an ASO that knocks down the antisense strand of *SMN* (called *SMN-AS1*).[57,58] Unlike these two compounds, the 5′ UTR ASO works by a transcription-independent mechanism, and we propose that the shift in splicing is due to SMN autoregulation.[40,41] The SMN complex is required for spliceosome biogenesis, and it is possible that the transcriptomic changes accompanying an increase in SMN protein levels include modulation of its own alternative splicing. This hypothesis is supported by our observation that there is no change in exon 7 inclusion in carrier fibroblasts treated with the 5′ UTR ASO, where baseline SMN levels (and spliceosome levels) are not low enough to perturb splicing.

Two papers have been published describing the antisense transcript, *SMN-AS1*.[58,59] The long noncoding RNA *SMN-AS1* is transcribed from *SMN* intron 1 but binds directly to the *SMN* transcription start site region. Here, it recruits PRC2 and reduces transcription of *SMN2*. Due to the proximity in binding locales, we wondered whether the 5′ UTR ASO reduces *SMN-AS1* activity. However, we found no difference in the transcription rate of *SMN2* with ASO treatment, indicating that the mechanism of action is likely independent of *SMN-AS1*.

Experiments are ongoing to elucidate the mechanism of action of the 5′ UTR ASO. We cannot yet rule out the possibility that the increase in SMN levels is due to off-target engagement, such as electrostatic interactions or sequence-specific interactions between the ASO and proteins. The determination of the mechanism of action of the 5′

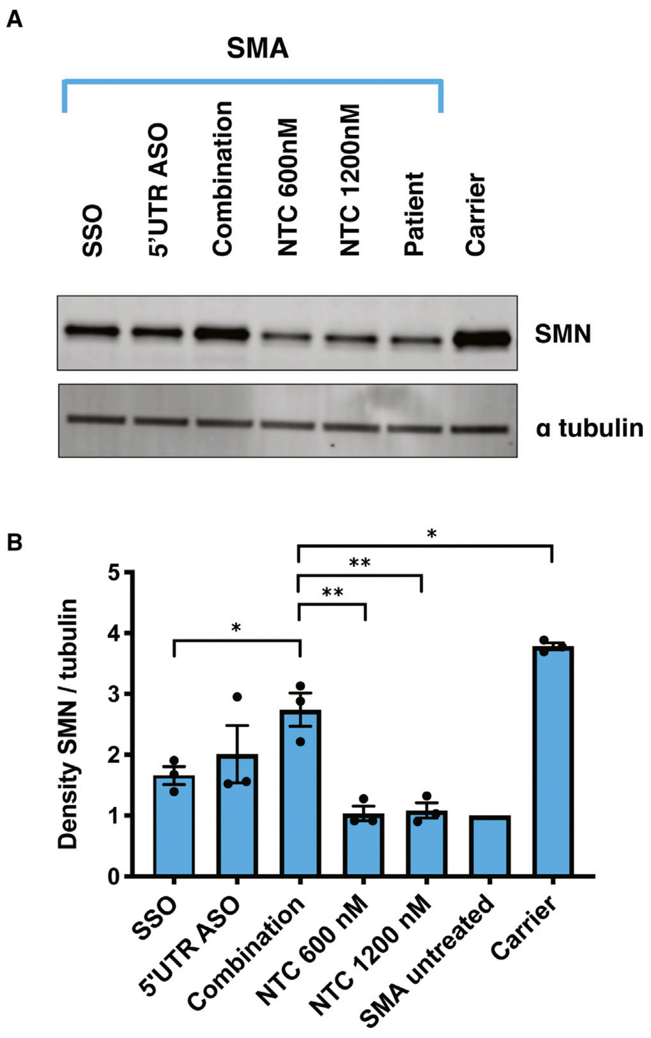

Figure 6. Use of a 5′ UTR ASO in combination with a SSO increases SMN protein levels more than use of the SSO alone

(A) SMA fibroblasts were transfected with the 2′-OMe 5′ UTR ASO (600 nM), the 2′-OMe SSO (600 nM), a combination of the two (1,200 nM total), or the 2′-OMe NTC ASO. 15 μg of protein was resolved per lane. (B) SMN levels were normalized to alpha tubulin, and expression was compared to levels in untreated SMA cells. Error bars show SEM. Statistical significance was determined by one-way ANOVA followed by Dunnett's test in comparison to combination. n = 3; $^{*}p < 0.05$; $^{**}p < 0.001$.

UTR ASO may reveal that there are other genes for which expression can be increased using a similar strategy. For example, the upregulation of utrophin via its 5′ UTR may be used for the treatment of Duchenne muscular dystrophy.[60] For now, our results add to the current understanding of SMN regulation and point toward a new therapeutic target for SMA.

MATERIALS AND METHODS

ASO synthesis

Three types of ASO were used in this study: (1) fully modified with 2′-OMe bases and phosphorothioate linkages; (2) fully modified with 2′-MOE bases and phosphorothioate linkages; and (3) PMOs. All 2′-OMe and 2′-MOE ASOs were purchased from Integrated DNA Technologies (IDT). PMOs were purchased from Gene Tools and subsequently conjugated to Pip9b2 as described previously.[61,62,65] ASO sequences are provided in Table S1.

Cell culture

SMA patient (Coriell; GM00232 and GM03813) and carrier (Coriell; GM03814) fibroblasts were cultured in Dulbecco's modified Eagle's medium (DMEM) supplemented with 15% fetal bovine serum (FBS) and maintained in a 37°C incubator with 5% CO_2. Fibroblast transfections were performed using RNAiMAX transfection reagent (Invitrogen). For each well of a 6-well plate, ASOs or small interfering RNA (siRNA) were complexed with 7.5 μL RNAiMAX in 300 μL Opti-MEM and added to cells at 70% confluency. The next day, media were changed to remove transfection reagents, and cells were harvested 2 days post-transfection (unless stated otherwise in the figure legends). The siRNAs used in these transfections were the following: DCP2 (Dharmacon; 167227), DCPS (Dharmacon; 28960), DXO (Dharmacon; 1797), NUDT3 s22028 (Thermo Fisher Scientific), NUDT16 (Dharmacon; 131870), SMN1/SMN2 (Thermo Fisher Scientific; s446415), XRN2 (Thermo Fisher Scientific; s22412), Negative Control No. 1 (Thermo Fisher Scientific; 4390843), and siGENOME Non-Targeting siRNA #2 (Dharmacon; D-001210-02-05).

HEK293T cells (ATCC) were cultured in DMEM, supplemented with 10% FBS, and maintained in a 37°C incubator with 5% CO_2. Plasmids were transfected using Lipofectamine 3000 (Invitrogen). Each well of a 6-well plate was transfected with 1 μg DNA, complexed with 3.75 μL Lipofectamine 3000 reagent and 4 μL P3000 reagent in 250 μL Opti-MEM. Media were changed 24 h later, and cells were harvested 2 days after transfection.

iPSCs were derived from fibroblasts grown from skin biopsies collected from type 2 or 3 SMA patients attending the Oxford Motor Neuron Disorders Clinic (under ethical approval granted by the South Wales Research Ethics Committee; ref 12/WA/0186). These were reprogrammed in the James Martin Stem Cell Facility, University of Oxford, using the method indicated in Table S2. Type I SMA iPSCs were a gift of Dr. Jeroen Pasterkamp, University of Utrecht. iPSCs were differentiated into motor neuron-like cells as described previously.[32,33] Briefly, the iPSCs were grown on Matrigel. They were then induced using equal volumes of DMEM/F12 and neurobasal medias supplemented with N2, B27, ascorbic acid (0.5 μM), 2-mercaptoethanol (50 μM), compound C (1 μM), and Chir99021 (3 μM). After 4 days in culture, media were further supplemented with retinoic acid (1 μM) and smoothened agonist (500 nM). The following day, the media were changed to media without compound C and Chir99021. The cells were then cultured for 4–5 additional days before being split 1:3 using Accutase. Rock inhibitor was added for 24 h. After splitting, the media were supplemented with growth factors brain-derived neurotrophic factor (BDNF; 10 μM), glial cell line-derived neurotrophic factor (GDNF; 10 μM), N-[N-(3,5-difluorophenacetyl)-L-ala-nyl]-S-phenylglycine t-butyl ester (DAPT) (10 mM),

and laminin (0.5 mg/mL) for 7 days. DAPT and laminin were then removed from the media, and the neurons remained in culture until day 28. Neurons were treated with ASOs (with no transfection reagents) on day 24 and again on day 26, before collection on day 28.

Cloning

To test the effect of the upstream start codon on gene expression, reporter constructs were created using the pBI-CMV4 bidirectional promoter vector (Takara Bio). The plasmid backbone was double digested with BglII and EcoRI and gel extracted using the NucleoSpin Gel and PCR Clean-Up Kit (Takara Bio). Then, DsRed2 was replaced with PCR-amplified mCherry using the In-Fusion HD Cloning Plus Kit (Takara Bio). Mini-prepped mCherry plasmid was double digested with BamHI and NotI for insertion of the reporter protein coding sequence. As a template for the reporter, we used a gBlocks Gene Fragment (IDT) containing the 5′ UTR of *SMN2* followed by the coding sequence of EGFP followed by exon 2 through exon 3 of the human beta-globin gene (*HBB*). The well-characterized *HBB* exon 2, intron 2, exon 3 splice junction was included to make the reporters more sensitive to endogenous gene regulation. This feature is important since one of the means through which uORFs downregulate gene expression is promoting nonsense-mediated decay, a process that requires the presence of an exon junction complex. The gBlocks Gene Fragment was PCR amplified using CloneAmp HiFi PCR Premix (Takara Bio) and 500 nM each primer, where the forward primer contained the desired uORF mutation(s). Primer sequences are provided in Table S7. One of the reporters (frame shift) required a second gBlocks Gene Fragment in order to obtain a plasmid with the desired mutation (Table S6). These PCR products and digested plasmids were gel extracted and cloned using the In-Fusion HD Cloning Plus Kit (Takara Bio), as above.

For all cloning work, One Shot TOP10 Chemically Competent *E. coli* (Invitrogen) was transformed with In-Fusion reaction products. Plasmids were extracted from bacterial cultures using the QIAprep Spin Miniprep Kit (QIAGEN) and tested by restriction enzyme digest. Selected clones were expanded and plasmids extracted using the Hi-Speed Plasmid Maxi Kit (QIAGEN). The sequences of inserted DNA fragments were verified in all plasmids by Sanger sequencing (GENEWIZ). Details of primers used for sequencing are provided in Table S8.

Immunoblotting

Lysates were prepared in radioimmunoprecipitation assay (RIPA) buffer (50 mM Tris-HCl, pH 8.0, 150 mM NaCl, 5 mM EDTA, 1% NP-40 [IGEPAL], 1% sodium deoxycholate, 0.1% sodium dodecyl sulfate [SDS]), supplemented with Halt Protease and Phosphatase Inhibitor Cocktail (Thermo Fisher Scientific) or cOmplete Protease Inhibitor Cocktail (Roche). After lysing on ice, samples were centrifuged (15 min, 14,000 × *g*, 4°C) to remove pelleted material. Protein concentrations were determined by the Bradford assay using Protein Assay Dye Reagent Concentrate (Bio-Rad). Samples were then prepared in RIPA and 4× sample loading buffer (H_2O, Tris-HCl, 40% glycerol, 0.08 g/mL SDS, 5% [v/v] 2-mercaptoethanol, bromophenol blue). Proteins were resolved on Novex 4%–20% Tris-Glycine WedgeWell Gels (Invitrogen) and transferred to a 0.45-μm polyvinylidene fluoride (PVDF) membrane. The amount of protein loaded per lane in micrograms is indicated in the relevant figure legend. Membranes were blocked with 5% (w/v) milk in Tris-buffered saline and 0.1% Tween (TBST) before incubation with primary antibodies at the indicated dilutions: mouse anti-SMN (BD Biosciences; 610647, 1:1,000 dilution), anti-Gemin6 (Abcam; ab88290, 1:500 dilution), anti-Gemin8 (Abcam; ab46778, 1:1,000 dilution), rabbit anti-alpha tubulin (Abcam, ab4074, 1:5,000 dilution; or Cell Signaling Technology, 2144, 1:2,000), rabbit anti-heat shock protein (HSP)90 (Cell Signaling Technology; 4874, 1:5,000 dilution), rabbit anti-GFP (Abcam; ab290, 1:10,000 dilution), and mouse anti-mCherry (Abcam; ab125096, 1:2,000 dilution). The membranes were then incubated with either IRDye or horseradish peroxidase (HRP) secondary antibodies and detected on a LI-COR Odyssey or a Bio-Rad ChemiDoc XRS+ imaging system, respectively. Primary antibodies and secondary antibodies were incubated overnight at 4°C or 1 h at room temperature with shaking and were followed by three washes with TBST. Densitometric analysis of protein signal was done using ImageJ software.

RNA stability assay

For mRNA stability assays, fibroblasts were transfected with 600 nM 2′-OMe ASOs as described above. 2 days post-transfection, fibroblasts were treated with media containing 5 μg/mL actinomycin D (Sigma-Aldrich). The cells were then collected in 0.5 mL TRIzol at the indicated time points after treatment with actinomycin D. After adding 100 μL chloroform, the samples were vortexed and centrifuged (15 min, 12,000 × *g*, 4°C). The supernatant was transferred to a new tube, to which 1.5 volumes of 100% ethanol were added. The samples were then pipetted into columns, and RNA purification continued according to the miRNeasy Mini Kit manual (QIAGEN). Total RNA was converted into cDNA using the High-Capacity cDNA Reverse Transcription Kit (Applied Biosystems). qRT-PCRs were performed in triplicate using the QuantStudio 6 Flex Real-Time PCR System (Applied Biosystems). 10 μL qRT-PCR reactions contained Power SYBR Green (Applied Biosystems) with 200 nM of each target primer and cDNA diluted in nuclease-free water (to a concentration for which primers have 90%–110% efficiency). Primer sequences are provided in Table S3.

Transcription assay

Transcription was measured using the Click-iT Nascent RNA Capture Kit (Invitrogen). In summary, 3 days after ASO transfection, SMA fibroblasts were pulsed with 0.5 mM EU for 1 h and then collected in 0.5 mL TRIzol. 100 μL chloroform was added to each sample, which was then vortexed and centrifuged (15 min, 12,000 × *g*, 4°C). The supernatant was transferred to a new tube, and 1.5 volumes of 100% ethanol were added. The samples were then pipetted into columns, and RNA purification continued according to the RNeasy Mini Kit manual (QIAGEN).

750 ng purified RNA was used in each Click reaction. Subsequently, 400 ng biotinylated RNA was mixed with 25 μL magnetic bead suspension for each binding reaction. After washing away unbound

RNA, on-bead cDNA synthesis was performed using the SuperScript VILO cDNA Synthesis Kit (Thermo Fisher Scientific) in a 50-μL final reaction volume. 500 ng total RNA (pre-biotinylation) was also converted to cDNA in a 15-μL reaction using the SuperScript VILO cDNA Synthesis Kit, which represents steady-state mRNA.

qRT-PCRs were performed as 20 μL reactions in triplicate. Reactions contained Power SYBR Green (Applied Biosystems), 200 nM of each target primer, 1 μL cDNA, and nuclease-free water. cDNA of total RNA was diluted 1:4 in nuclease-free water, whereas cDNA of biotinylated RNA was undiluted. qRT-PCR plates were run on the StepOnePlus Real-Time PCR System (Applied Biosystems).

Splice isoform analysis

SMN2 exon 7 splicing was qualitatively assessed by gel electrophoresis of RT-PCR products. First, RNA was extracted, and cDNA was converted according to the protocol described above for the RNA stability assay. The cDNA from ASO-treated samples was used as template for PCR. The reaction was performed with PCR Master (Roche) and with primers situated in exon 5 and exon 8 of *SMN2*. Primer sequences are provided in Table S4. Amplicons were resolved on 2% agarose gels. To ensure that the full-length amplicon did not sequester the Δ7 amplicon, a test run was performed in which samples were diluted, denatured by heating to 95°C for 5 min, and then immediately placed on ice until being loaded on a gel. The results were consistent between heated and unheated samples, so the extra denaturing and steps were omitted. Exon 7 splicing was quantitatively assessed for the same samples using the qRT-PCR protocol described for the RNA stability assay.

Bioinformatics

Ribosome profiling sequencing data from GEO sample accessions (GEO: GSM1047584, GSM1047585, and GSM3566399) were downloaded in the fastq format using the fastq-dump command supported in the NCBI SRA Toolkit (https://ncbi.github.io/sra-tools/). The FastQC tool (https://www.bioinformatics.babraham.ac.uk/projects/fastqc/) was used to inspect the quality of the sequence data. To clip adaptor sequences that may be present and to remove low-quality sequences, the Trimmomatic tool (http://www.usadellab.org/cms/?page=trimmomatic) was used with the following command-line specifications: "ILLUMINACLIP:TruSeq3-SE-2.fa:2:30:10 HEADCROP:11 TRAILING:20 SLIDINGWINDOW:4:20 MINLEN:15." Reads were mapped to a custom reference genome. The custom reference genome was constructed by deleting all *SMN* gene annotations from human reference genome hg19 and then, to the modified hg19, adding the 215P15 clone sequence as a separate contig, as described previously.[44] For reference mapping against the customized version of the human genome, the RNA sequencing (RNA-Seq) tool, supported in the CLCbio Genomics Workbench (version [v.]12) was used under default parameters. Mapped reads were visualized using Integrative Genomics Viewer (IGV) version 2.4.8 (http://software.broadinstitute.org/software/igv/).

MEF isolation and culture

Mouse work was performed in the Biomedical Sciences Unit at the University of Oxford as authorized by the UK Home Office (Animal Scientific Procedures Act 1986). Taiwanese SMA mice were bred and maintained as described on The Jackson Laboratory website and as described previously.[36,63] MEFs were isolated from strain FVB.Cg-*Smn1*^{tm1}*Hung*Tg(SMN2)2Hung/J (Jackson Laboratory; 005058), crossed with strain FVB.129P2(B6)-*Smn1*^{tm1Hung}/J (Jackson Laboratory; 031678), using a method described previously.[64]

After 2 additional days of culturing in MEF culture medium, the cells were plated for ASO transfection. For the MEF lines with sufficient cell counts, duplicate wells were plated (one for transfection with the 5′ UTR ASO and one for transfection with the NTC ASO). Single wells were plated for the MEF lines with fewer cells. The MEFs were transfected with ASO using RNAi MAX as described above. 3 days later, the MEFs were collected in RIPA buffer and subsequently assayed by immunoblotting.

KO/D7;SMN2- and *KO/F7*-immortalized MEFs[37] shared by the Burghes lab were transfected with ASO and immunoblotted 2 days post-transfection.

Statistics

Data were analyzed in Microsoft Excel and GraphPad Prism 8. In experiments with an untreated condition, all sample values are shown as a fold difference relative to the untreated samples. For these figures, error bars show standard error of the mean (SEM) of the fold differences. One-way ANOVA with Dunnett's multiple comparisons test was used to determine statistical significance.

In experiments without an untreated condition, all samples were expressed as a fold difference relative to the NTC samples. For these figures, error bars show propagated error. In short, when normalizing the protein of interest to the loading control, the error associated with each signal intensity value was divided by the mean signal intensity value, and this fraction was squared. The values for the two proteins were summed, and then the square root was calculated. The resulting error value was used in a second, identical round of error computation in order to propagate it through the fold-difference calculation. Statistical significance was determined by t test between the normalized signal intensity values for the two sample groups.

For the RNA stability assay, we used a linear mixed model with total *SMN* mRNA level as the outcome variable and hours post-actinomycin D treatment, group, and their interaction as independent variables. Sample was specified as a random intercept. The significant interaction statistic was tested using a likelihood ratio test between the full model and a reduced no-interaction model and reported using a chi-square statistic and p value. If significant, the slopes for each group were then compared to each other and were Bonferroni corrected for 3 comparisons.

Data presentation

Graphs were made using GraphPad Prism 8. Some schematics were created using BioRender (BioRender.com). Figures were assembled in Microsoft PowerPoint and Adobe Illustrator.

SUPPLEMENTAL INFORMATION

Supplemental Information can be found online at https://doi.org/10.1016/j.omtn.2020.12.027.

ACKNOWLEDGMENTS

The authors would like to thank Corey Ruhno for kindly sharing his custom reference genome with us. We also thank the lab of Arthur Burghes for sharing the *KO/D7;SMN2* and *KO/F7* mouse embryonic fibroblasts. We would like to thank Gina Norato for statistical analysis of the RNA stability data. We thank Keely Winkelsas for considerable laboratory assistance and also thank Marcus Hafner, Roy Parker, Gideon Dreyfuss, Frank Bennett, Frank Rigo, and Charlotte Sumner for helpful discussions. A.M.W. was an NIH-Oxford-Cambridge Scholars Program graduate student. This work was supported by intramural research funds from NINDS, as well as funding from the Gwendolyn Strong Foundation, SMA UK, Wellcome Trust, and Medical Research Council.

AUTHOR CONTRIBUTIONS

A.M.W., C.G., K.H.F., S.A., K.T., S.M.H., and C.R. designed the experiments. M.B. helped in experimental set-up. A.M.W., G.G.H., K.C., S.M.H., and S.A. conducted the experiments. A.M.W., K.H.F., C.G., K.J., C.R., and M.J.W. analyzed and interpreted the data. A.M.W. wrote the first draft, and K.H.F., C.G., K.T., S.M.H., S.A., and C.R. contributed to the final version of the manuscript.

DECLARATION OF INTERESTS

A patent application was filed for the 5′ UTR ASOs described in this manuscript. M.J.W. is a founder of and shareholder in PepGen.

REFERENCES

1. Lefebvre, S., Bürglen, L., Reboullet, S., Clermont, O., Burlet, P., Viollet, L., Benichou, B., Cruaud, C., Millasseau, P., Zeviani, M., et al. (1995). Identification and characterization of a spinal muscular atrophy-determining gene. Cell *80*, 155–165.

2. Crawford, T.O., and Pardo, C.A. (1996). The neurobiology of childhood spinal muscular atrophy. Neurobiol. Dis. *3*, 97–110.

3. Pellizzoni, L., Yong, J., and Dreyfuss, G. (2002). Essential role for the SMN complex in the specificity of snRNP assembly. Science *298*, 1775–1779.

4. Kolb, S.J., Battle, D.J., and Dreyfuss, G. (2007). Molecular functions of the SMN complex. J. Child Neurol. *22*, 990–994.

5. Rossoll, W., Jablonka, S., Andreassi, C., Kröning, A.K., Karle, K., Monani, U.R., and Sendtner, M. (2003). Smn, the spinal muscular atrophy-determining gene product, modulates axon growth and localization of β-actin mRNA in growth cones of motoneurons. J. Cell Biol. *163*, 801–812.

6. Akten, B., Kye, M.J., Hao, T., Wertz, M.H., Singh, S., Nie, D., Huang, J., Merianda, T.T., Twiss, J.L., Beattie, C.E., et al. (2011). Interaction of survival of motor neuron (SMN) and HuD proteins with mRNA cpg15 rescues motor neuron axonal deficits. Proc. Natl. Acad. Sci. USA *108*, 10337–10342.

7. Fallini, C., Donlin-Asp, P.G., Rouanet, J.P., Bassell, G.J., and Rossoll, W. (2016). Deficiency of the survival of motor neuron protein impairs mRNA localization and local translation in the growth cone of motor neurons. J. Neurosci. *36*, 3811–3820.

8. Rage, F., Boulisfane, N., Rihan, K., Neel, H., Gostan, T., Bertrand, E., Bordonné, R., and Soret, J. (2013). Genome-wide identification of mRNAs associated with the protein SMN whose depletion decreases their axonal localization. RNA *19*, 1755–1766.

9. Schrank, B., Götz, R., Gunnersen, J.M., Ure, J.M., Toyka, K.V., Smith, A.G., and Sendtner, M. (1997). Inactivation of the survival motor neuron gene, a candidate gene for human spinal muscular atrophy, leads to massive cell death in early mouse embryos. Proc. Natl. Acad. Sci. USA *94*, 9920–9925.

10. Calucho, M., Bernal, S., Alías, L., March, F., Venceslá, A., Rodríguez-Álvarez, F.J., Aller, E., Fernández, R.M., Borrego, S., Millán, J.M., et al. (2018). Correlation between SMA type and SMN2 copy number revisited: An analysis of 625 unrelated Spanish patients and a compilation of 2834 reported cases. Neuromuscul. Disord. *28*, 208–215.

11. Wirth, B., Brichta, L., Schrank, B., Lochmüller, H., Blick, S., Baasner, A., and Heller, R. (2006). Mildly affected patients with spinal muscular atrophy are partially protected by an increased SMN2 copy number. Hum. Genet. *119*, 422–428.

12. Lorson, C.L., Hahnen, E., Androphy, E.J., and Wirth, B. (1999). A single nucleotide in the SMN gene regulates splicing and is responsible for spinal muscular atrophy. Proc. Natl. Acad. Sci. USA *96*, 6307–6311.

13. Monani, U.R., Lorson, C.L., Parsons, D.W., Prior, T.W., Androphy, E.J., Burghes, A.H.M., and McPherson, J.D. (1999). A single nucleotide difference that alters splicing patterns distinguishes the SMA gene SMN1 from the copy gene SMN2. Hum. Mol. Genet. *8*, 1177–1183.

14. Cartegni, L., Hastings, M.L., Calarco, J.A., de Stanchina, E., and Krainer, A.R. (2006). Determinants of exon 7 splicing in the spinal muscular atrophy genes, SMN1 and SMN2. Am. J. Hum. Genet. *78*, 63–77.

15. Mailman, M.D., Heinz, J.W., Papp, A.C., Snyder, P.J., Sedra, M.S., Wirth, B., Burghes, A.H.M., and Prior, T.W. (2002). Molecular analysis of spinal muscular atrophy and modification of the phenotype by SMN2. Genet. Med. *4*, 20–26.

16. Crawford, T.O., Paushkin, S.V., Kobayashi, D.T., Forrest, S.J., Joyce, C.L., Finkel, R.S., Kaufmann, P., Swoboda, K.J., Tiziano, D., Lomastro, R., et al.; Pilot Study of Biomarkers for Spinal Muscular Atrophy Trial Group (2012). Evaluation of SMN protein, transcript, and copy number in the biomarkers for spinal muscular atrophy (BforSMA) clinical study. PLoS ONE *7*, e33572.

17. Mendell, J.R., Al-Zaidy, S., Shell, R., Arnold, W.D., Rodino-Klapac, L.R., Prior, T.W., Lowes, L., Alfano, L., Berry, K., Church, K., et al. (2017). Single-dose gene-replacement therapy for spinal muscular atrophy. N. Engl. J. Med. *377*, 1713–1722.

18. Finkel, R.S., Mercuri, E., Darras, B.T., Connolly, A.M., Kuntz, N.L., Kirschner, J., Chiriboga, C.A., Saito, K., Servais, L., Tizzano, E., et al.; ENDEAR Study Group (2017). Nusinersen versus sham control in infantile-onset spinal muscular atrophy. N. Engl. J. Med. *377*, 1723–1732.

19. Finkel, R.S., Chiriboga, C.A., Vajsar, J., Day, J.W., Montes, J., De Vivo, D.C., Yamashita, M., Rigo, F., Hung, G., Schneider, E., et al. (2016). Treatment of infantile-onset spinal muscular atrophy with nusinersen: a phase 2, open-label, dose-escalation study. Lancet *388*, 3017–3026.

20. Hua, Y., Vickers, T.A., Okunola, H.L., Bennett, C.F., and Krainer, A.R. (2008). Antisense masking of an hnRNP A1/A2 intronic splicing silencer corrects SMN2 splicing in transgenic mice. Am. J. Hum. Genet. *82*, 834–848.

21. Ratni, H., Ebeling, M., Baird, J., Bendels, S., Bylund, J., Chen, K.S., Denk, N., Feng, Z., Green, L., Guerard, M., et al. (2018). Discovery of risdiplam, a selective survival of motor neuron-2 (SMN2) gene splicing modifier for the treatment of spinal muscular atrophy (SMA). J. Med. Chem. *61*, 6501–6517.

22. Campagne, S., Boigner, S., Rüdisser, S., Moursy, A., Gillioz, L., Knörlein, A., Hall, J., Ratni, H., Cléry, A., and Allain, F.H.T. (2019). Structural basis of a small molecule targeting RNA for a specific splicing correction. Nat. Chem. Biol. *15*, 1191–1198.

23. Mignone, F., Gissi, C., Liuni, S., and Pesole, G. (2002). Untranslated regions of mRNAs. Genome Biol. *3*, reviews0004.1—reviews0004.10.

24. Chatterjee, S., and Pal, J.K. (2009). Role of 5′- and 3′-untranslated regions of mRNAs in human diseases. Biol. Cell *101*, 251–262.

25. Monani, U.R., McPherson, J.D., and Burghes, A.H.M. (1999). Promoter analysis of the human centromeric and telomeric survival motor neuron genes (SMNC and SMNT). Biochim. Biophys. Acta *1445*, 330–336.

26. Germain-Desprez, D., Brun, T., Rochette, C., Semionov, A., Rouget, R., and Simard, L.R. (2001). The SMN genes are subject to transcriptional regulation during cellular differentiation. Gene *279*, 109–117.

27. Echaniz-Laguna, A., Miniou, P., Bartholdi, D., and Melki, J. (1999). The promoters of the survival motor neuron gene (SMN) and its copy (SMNc) share common regulatory elements. Am. J. Hum. Genet. *64*, 1365–1370.

28. Calvo, S.E., Pagliarini, D.J., and Mootha, V.K. (2009). Upstream open reading frames cause widespread reduction of protein expression and are polymorphic among humans. Proc. Natl. Acad. Sci. USA *106*, 7507–7512.

29. Liang, X.H., Shen, W., Sun, H., Migawa, M.T., Vickers, T.A., and Crooke, S.T. (2016). Translation efficiency of mRNAs is increased by antisense oligonucleotides targeting upstream open reading frames. Nat. Biotechnol. *34*, 875–880.

30. Rouleau, S.G., Beaudoin, J.D., Bisaillon, M., and Perreault, J.P. (2015). Small antisense oligonucleotides against G-quadruplexes: specific mRNA translational switches. Nucleic Acids Res. *43*, 595–606.

31. Liang, X.H., Sun, H., Shen, W., Wang, S., Yao, J., Migawa, M.T., Bui, H.-H., Damle, S.S., Riney, S., Graham, M.J., et al. (2017). Antisense oligonucleotides targeting translation inhibitory elements in 5′ UTRs can selectively increase protein levels. Nucleic Acids Res. *45*, 9528–9546.

32. Dafinca, R., Scaber, J., Ababneh, N., Lalic, T., Weir, G., Christian, H., Vowles, J., Douglas, A.G.L., Fletcher-Jones, A., Browne, C., et al. (2016). C9orf72 hexanucleotide expansions are associated with altered endoplasmic reticulum calcium homeostasis and stress granule formation in induced pluripotent stem cell-derived neurons from patients with amyotrophic lateral sclerosis and frontotemporal demen. Stem Cells *34*, 2063–2078.

33. Ababneh, N.A., Scaber, J., Flynn, R., Douglas, A., Barbagallo, P., Candalija, A., Turner, M.R., Sims, D., Dafinca, R., Cowley, S.A., and Talbot, K. (2020). Correction of amyotrophic lateral sclerosis related phenotypes in induced pluripotent stem cell-derived motor neurons carrying a hexanucleotide expansion mutation in C9orf72 by CRISPR/Cas9 genome editing using homology-directed repair. Hum. Mol. Genet. *29*, 2200–2217.

34. Schoch, K.M., and Miller, T.M. (2017). Antisense oligonucleotides: translation from mouse models to human neurodegenerative diseases. Neuron *94*, 1056–1070.

35. Shen, X., and Corey, D.R. (2018). Chemistry, mechanism and clinical status of antisense oligonucleotides and duplex RNAs. Nucleic Acids Res. *46*, 1584–1600.

36. Hsieh-Li, H.M., Chang, J.G., Jong, Y.J., Wu, M.H., Wang, N.M., Tsai, C.H., and Li, H. (2000). A mouse model for spinal muscular atrophy. Nat. Genet. *24*, 66–70.

37. Blatnik, A.J., McGovern, V.L., Le, T.T., Iyer, C.C., Kaspar, B.K., and Burghes, A.H.M. (2021). Conditional deletion of SMN in cell culture identifies functional SMN alleles. Hum. Mol. Genet. *29*, 3477–3492.

38. Monani, U.R., Sendtner, M., Coovert, D.D., Parsons, D.W., Andreassi, C., Le, T.T., Jablonka, S., Schrank, B., Rossoll, W., Prior, T.W., et al. (2000). The human centromeric survival motor neuron gene (SMN2) rescues embryonic lethality in Smn(-/-) mice and results in a mouse with spinal muscular atrophy. Hum. Mol. Genet. *9*, 333–339.

39. Gabanella, F., Butchbach, M.E.R., Saieva, L., Carissimi, C., Burghes, A.H.M., and Pellizzoni, L. (2007). Ribonucleoprotein assembly defects correlate with spinal muscular atrophy severity and preferentially affect a subset of spliceosomal snRNPs. PLoS ONE *2*, e921.

40. Jodelka, F.M., Ebert, A.D., Duelli, D.M., and Hastings, M.L. (2010). A feedback loop regulates splicing of the spinal muscular atrophy-modifying gene, SMN2. Hum. Mol. Genet. *19*, 4906–4917.

41. Ruggiu, M., McGovern, V.L., Lotti, F., Saieva, L., Li, D.K., Kariya, S., Monani, U.R., Burghes, A.H.M., and Pellizzoni, L. (2012). A role for SMN exon 7 splicing in the selective vulnerability of motor neurons in spinal muscular atrophy. Mol. Cell. Biol. *32*, 126–138.

42. Loayza-Puch, F., Drost, J., Rooijers, K., Lopes, R., Elkon, R., and Agami, R. (2013). p53 induces transcriptional and translational programs to suppress cell proliferation and growth. Genome Biol. *14*, R32.

43. Martinez, T.F., Chu, Q., Donaldson, C., Tan, D., Shokhirev, M.N., and Saghatelian, A. (2020). Accurate annotation of human protein-coding small open reading frames. Nat. Chem. Biol. *16*, 458–468.

44. Ruhno, C., McGovern, V.L., Avenarius, M.R., Snyder, P.J., Prior, T.W., Nery, F.C., Muhtaseb, A., Roggenbuck, J.S., Kissel, J.T., Sansone, V.A., et al. (2019). Complete sequencing of the SMN2 gene in SMA patients detects SMN gene deletion junctions and variants in SMN2 that modify the SMA phenotype. Hum. Genet. *138*, 241–256.

45. Chang, J.-G., Hsieh-Li, H.-M., Jong, Y.-J., Wang, N.M., Tsai, C.-H., and Li, H. (2001). Treatment of spinal muscular atrophy by sodium butyrate. Proc. Natl. Acad. Sci. USA *98*, 9808–9813.

46. Sumner, C.J., Huynh, T.N., Markowitz, J.A., Perhac, J.S., Hill, B., Coovert, D.D., Schussler, K., Chen, X., Jarecki, J., Burghes, A.H.M., et al. (2003). Valproic acid increases SMN levels in spinal muscular atrophy patient cells. Ann. Neurol. *54*, 647–654.

47. Avila, A.M., Burnett, B.G., Taye, A.A., Gabanella, F., Knight, M.A., Hartenstein, P., Cizman, Z., Di Prospero, N.A., Pellizzoni, L., Fischbeck, K.H., and Sumner, C.J. (2007). Trichostatin A increases SMN expression and survival in a mouse model of spinal muscular atrophy. J. Clin. Invest. *117*, 659–671.

48. Butchbach, M.E.R., Lumpkin, C.J., Harris, A.W., Saieva, L., Edwards, J.D., Workman, E., Simard, L.R., Pellizzoni, L., and Burghes, A.H.M. (2016). Protective effects of butyrate-based compounds on a mouse model for spinal muscular atrophy. Exp. Neurol. *279*, 13–26.

49. Liu, H., Yazdani, A., Murray, L.M., Beauvais, A., and Kothary, R. (2014). The Smn-independent beneficial effects of trichostatin A on an intermediate mouse model of spinal muscular atrophy. PLoS ONE *9*, e101225.

50. Le, T.T., Pham, L.T., Butchbach, M.E.R., Zhang, H.L., Monani, U.R., Coovert, D.D., Gavrilina, T.O., Xing, L., Bassell, G.J., and Burghes, A.H.M. (2005). SMNDelta7, the major product of the centromeric survival motor neuron (SMN2) gene, extends survival in mice with spinal muscular atrophy and associates with full-length SMN. Hum. Mol. Genet. *14*, 845–857.

51. Kwon, D.Y., Dimitriadi, M., Terzic, B., Cable, C., Hart, A.C., Chitnis, A., Fischbeck, K.H., and Burnett, B.G. (2013). The E3 ubiquitin ligase mind bomb 1 ubiquitinates and promotes the degradation of survival of motor neuron protein. Mol. Biol. Cell *24*, 1863–1871.

52. Jarecki, J., Chen, X., Bernardino, A., Coovert, D.D., Whitney, M., Burghes, A., Stack, J., and Pollok, B.A. (2005). Diverse small-molecule modulators of SMN expression found by high-throughput compound screening: early leads towards a therapeutic for spinal muscular atrophy. Hum. Mol. Genet. *14*, 2003–2018.

53. Butchbach, M.E.R., Singh, J., Thorsteinsdóttir, M., Saieva, L., Slominski, E., Thurmond, J., Andrésson, T., Zhang, J., Edwards, J.D., Simard, L.R., et al. (2010). Effects of 2,4-diaminoquinazoline derivatives on SMN expression and phenotype in a mouse model for spinal muscular atrophy. Hum. Mol. Genet. *19*, 454–467.

54. Gogliotti, R.G., Cardona, H., Singh, J., Bail, S., Emery, C., Kuntz, N., Jorgensen, M., Durens, M., Xia, B., Barlow, C., et al. (2013). The DcpS inhibitor RG3039 improves survival, function and motor unit pathologies in two SMA mouse models. Hum. Mol. Genet. *22*, 4084–4101.

55. Van Meerbeke, J.P., Gibbs, R.M., Plasterer, H.L., Miao, W., Feng, Z., Lin, M.Y., Rucki, A.A., Wee, C.D., Xia, B., Sharma, S., et al. (2013). The DcpS inhibitor RG3039 improves motor function in SMA mice. Hum. Mol. Genet. *22*, 4074–4083.

56. Gopalsamy, A., Narayanan, A., Liu, S., Parikh, M.D., Kyne, R.E., Jr., Fadeyi, O., Tones, M.A., Cherry, J.J., Nabhan, J.F., LaRosa, G., et al. (2017). Design of potent mRNA decapping scavenger enzyme (DcpS) inhibitors with improved physicochemical properties to investigate the mechanism of therapeutic benefit in spinal muscular atrophy (SMA). J. Med. Chem. *60*, 3094–3108.

57. Riessland, M., Brichta, L., Hahnen, E., and Wirth, B. (2006). The benzamide M344, a novel histone deacetylase inhibitor, significantly increases SMN2 RNA/protein levels in spinal muscular atrophy cells. Hum. Genet. *120*, 101–110.

58. d'Ydewalle, C., Ramos, D.M., Pyles, N.J., Ng, S.Y., Gorz, M., Pilato, C.M., Ling, K., Kong, L., Ward, A.J., Rubin, L.L., et al. (2017). The antisense transcript SMN-AS1 regulates SMN expression and is a novel therapeutic target for spinal muscular atrophy. Neuron *93*, 66–79.

59. Woo, C.J., Maier, V.K., Davey, R., Brennan, J., Li, G., Brothers, J., 2nd, Schwartz, B., Gordo, S., Kasper, A., Okamoto, T.R., et al. (2017). Gene activation of SMN by selective disruption of lncRNA-mediated recruitment of PRC2 for the treatment of spinal muscular atrophy. Proc. Natl. Acad. Sci. USA *114*, E1509–E1518.

60. Shieh, P.B. (2018). Emerging strategies in the treatment of Duchenne muscular dystrophy. Neurotherapeutics *15*, 840–848.

61. Betts, C., Saleh, A.F., Arzumanov, A.A., Hammond, S.M., Godfrey, C., Coursindel, T., Gait, M.J., and Wood, M.J. (2012). Pip6-PMO, a new generation of peptide-oligonucleotide conjugates with improved cardiac exon skipping activity for DMD treatment. Mol. Ther. Nucleic Acids *1*, e38.

62. van Westering, T.L.E., Lomonosova, Y., Coenen-Stass, A.M.L., Betts, C.A., Bhomra, A., Hulsker, M., Clark, L.E., McClorey, G., Aartsma-Rus, A., van Putten, M., et al.

(2020). Uniform sarcolemmal dystrophin expression is required to prevent extracellular microRNA release and improve dystrophic pathology. J. Cachexia Sarcopenia Muscle *11*, 578–593.

63. Gogliotti, R.G., Hammond, S.M., Lutz, C., and Didonato, C.J. (2010). Molecular and phenotypic reassessment of an infrequently used mouse model for spinal muscular atrophy. Biochem. Biophys. Res. Commun. *391*, 517–522.

64. Xu, J. (2005). Preparation, culture, and immortalization of mouse embryonic fibroblasts. Curr. Protoc. Mol. Biol., Chapter 28, Unit 28.1.

65. Klein, A.F., Varela, M.A., Arandel, L., Holland, A., Naouar, N., Arzumanov, A., Seoane, D., Revillod, L., Bassez, G., Ferry, A., et al. (2019). Peptide-conjugated oligonucleotides evoke long-lasting myotonic dystrophy correction in patient-derived cells and mice. J. Clin. Invest. *129*, 4739–4744.

OMTN, Volume *23*

Supplemental Information

Targeting the 5′ untranslated region of *SMN2* as a therapeutic strategy for spinal muscular atrophy

Audrey M. Winkelsas, Christopher Grunseich, George G. Harmison, Katarzyna Chwalenia, Carlo Rinaldi, Suzan M. Hammond, Kory Johnson, Melissa Bowerman, Sukrat Arya, Kevin Talbot, Matthew J. Wood, and Kenneth H. Fischbeck

Table S1. Properties of ASOs used in experiments.

The first 7 ASOs listed were fully modified with 2’-O-methyl (2’OMe) bases and phosphorothioate (PS) linkages. The non-targeting control (NTC) ASO uses the “Standard Control” sequence which was described by Gene Tools, LLC as having no RNA target and minimal biological activity. The middle 3 ASOs were fully modified with 2’-O-methoxyethyl (2’-MOE) bases and phosphorothioate (PS) linkages. The final 2 ASOs were in the phosphorodiamidate morpholino oligomer (PMO) chemistry.

Sequence name	ASO sequence (5' -> 3')	Chemistry	% GC
ASO #1	GUUAUCGCCCUCCCACAUUUGUGG	2’-OMe	54.2
ASO #2	UGGUUAUCGCCCUCCCACAUUUGU	2’-OMe	50.0
ASO #3	AGUGGUUAUCGCCCUCCCACAUUU	2’-OMe	50.0
ASO #4	CGAGUGGUUAUCGCCCUCCCACAU	2’-OMe	58.3
ASO #5	UACGAGUGGUUAUCGCCCUCCCAC	2’-OMe	58.3
ASO #6	UUCUGGGAGCGGAACAGUACGGUG	2’-OMe	58.3
NTC	CCUCUUACCUCAGUUACAAUUUAUA	2’-OMe	32.0
5’UTR ASO	GUUAUCGCCCUCCCACAUUUGUGG	2’-MOE	54.2
NTC	CCUCUUACCUCAGUUACAAUUUAUA	2’-MOE	32.0
Scrambled	GUGGUCGCAUUUCCUCGUUACCAC	2’-MOE	54.2
5’UTR pPMO	TGGTTATCGCCCTCCCACATTTGTG	PMO	52.0
DM1 pPMO[63]	CAGCAGCAGCAGCAGCAGCAG	PMO	66.7%

Table S2. SMA iPSC lines used for motor neuron differentiation.

iPSC line name	Gender	Age at biopsy	Reprogramming method	Genotype	Disease
SMA A4	F	109 days	STEMCCA Lentivirus Reprogramming	Homozygous *SMN1* deletion, 2 copies *SMN2*	SMA Type 1
RS 1.2	M	74 days	STEMCCA Lentivirus Reprogramming		Age-matched control
IPS-OXSMA-03 (Clone 03 03)	M	49 years	CytoTune Sendai Reprogramming	Homozygous *SMN1* deletion, 3 copies *SMN2*	SMA Type 2
IPS-OXSMA-02 (Clone 02 04)	M	30 years	CytoTune Sendai Reprogramming	Homozygous *SMN1* deletion, 4 copies *SMN2*	SMA Type 3
OX1 (Clone 841-03-01)	M	36 years	CytoTune Sendai Reprogramming		Age-matched control

Table S3. Primers for gene expression analysis by qRT-PCR.

RNA	Primer name	Primer sequences (5’ -> 3’)	Chemistry	T_a (ºC)
FL-SMN	FL-SMN qF	GCTTTGGGAAGTATGTTAATTTCA	Sybr Green	60
	FL-SMN qR	CTATGCCAGCATTTCTCCTTAATT		
SMNΔ7	SMNΔ7 qF	CCACCACCCCACTTACTATCA	Sybr Green	60
	SMNΔ7 qR	GCTCTATGCCAGCATTTCCATA		
Total SMN	Total SMN qF	GCGATGATTCTGACATTTGG	Sybr Green	60
	Total SMN qR	GGAAGCTGCAGTATTCTTCT		
GAPDH	GAPDH qF	CTCAACGACCACTTTGTCAAGCTC	Sybr Green	60
	GAPDH qR	TCTTACTCCTTGGAGGCCATGT		

Table S4. Primers for *SMN2* exon 7 inclusion RT-PCR.

Primer name	Primer sequences (5’ -> 3’)	T_a (ºC)
SMN PAGE Fwd	AGGTCTAAAATTCAATGGCCCA	60
SMN PAGE Rev	GTGTCATTTAGTGCTGCTCTATGC	

Table S5. Confirming Click-iT assay specificity to nascent RNA.

Before the 5-ethynyl uridine pulse, fibroblasts were treated with actinomycin D to prevent transcription and serve as an experimental control. Those samples have higher qRT-PCR C_t values (less amplification of *SMN* and *GAPDH*) than samples not treated with actinomycin D. Values shown are mean plus or minus standard deviation. n = 3.

	Average C_t *SMN*	Average C_t *GAPDH*
NTC ASO	27.41 ± 0.46	25.94 ± 0.08
5'UTR ASO	26.49 ± 0.81	25.40 ± 0.62
Untreated	27.20 ± 0.73	25.99 ± 0.61
Actinomycin D	34.04 ± 0.60	29.99 ± 0.39
Carrier	25.47 ± 0.05	25.92 ± 0.21

Table S6. Double-stranded DNA fragments used in plasmid construction.

All sequences purchased as gBlocks Gene Fragments or Ultramer DNA Oligos from IDT. Lower case letters indicate intronic sequence.

**DNA name**	**Sequence (5' -> 3')**
gBlocks: *SMN2* 5'UTR + eGFP + *HBB*	CCACAAATGTGGGAGGGCGATAACCACTCGTAGAAAGCGTGAGAAGTTACT<br>ACAAGCGGTCCTCCCGGCCACCGTACTGTTCCGCTCCCAGAAGCCCCGGGC<br>GGCGGAAGTCGTCACTCTTAAGAAGGGACGGGGCCCCACGCTGCGCACCCG<br>CGGGTTTGCTATGGTGAGCAAGGGCGAGGAGCTGTTCACCGGGGTGGTGCC<br>CATCCTGGTCGAGCTGGACGGCGACGTAAACGGCCACAAGTTCAGCGTGTC<br>CGGCGAGGGCGAGGGCGATGCCACCTACGGCAAGCTGACCCTGAAGTTCAT<br>CTGCACCACCGGCAAGCTGCCCGTGCCCTGGCCCACCCTCGTGACCACCCTG<br>ACCTACGGCGTGCAGTGCTTCAGCCGCTACCCCGACCACATGAAGCAGCAC<br>GACTTCTTCAAGTCCGCCATGCCCGAAGGCTACGTCCAGGAGCGCACCATC<br>TTCTTCAAGGACGACGGCAACTACAAGACCCGCGCCGAGGTGAAGTTCGAG<br>GGCGACACCCTGGTGAACCGCATCGAGCTGAAGGGCATCGACTTCAAGGAG<br>GACGGCAACATCCTGGGGCACAAGCTGGAGTACAACTACAACAGCCACAA<br>CGTCTATATCATGGCCGACAAGCAGAAGAACGGCATCAAGGTGAACTTCAA<br>GATCCGCCACAACATCGAGGACGGCAGCGTGCAGCTCGCCGACCACTACCA<br>GCAGAACACCCCCATCGGCGACGGCCCCGTGCTGCTGCCCGACAACCACTA<br>CCTGAGCACCCAGTCCGCCCTGAGCAAAGACCCCAACGAGAAGCGCGATCA<br>CATGGTCCTGCTGGAGTTCGTGACCGCCGCCGGGATCACTCTCGGCATGGA<br>CGAGCTGTACAAGCTGCTGGTGGTCTACCCTTGGACCCAGAGGTTCTTTGAG<br>TCCTTTGGGGATCTGTCCACTCCTGATGCTGTTATGGGCAACCCTAAGGTGA<br>AGGCTCATGGCAAGAAAGTGCTCGGTGCCTTTAGTGATGGCCTGGCTCACC<br>TGGACAACCTCAAGGGCACCTTTGCCACACTGAGTGAGCTGCACTGTGACA<br>AGCTGCACGTGGATCCTGAGAACTTCAGGgtgagtctatgggacgcttgatgttttctttccccttctt<br>ttctatggttaagttcatgtcataggaaggggataagtaacagggtacagtttagaatgggaaacagacgaatgattgcatcagt<br>gtggaagtctcaggatcgttttagtttcttttatttgctgttcataacaattgttttcttttgtttaattcttgctttcttttttttcttctccgca<br>atttttactattatacttaatgccttaacattgtgtataacaaaaggaaatatctctgagatacattaagtaacttaaaaaaaaactttta<br>cacagtctgcctagtacattactatttggaatatatgtgtgcttatttgcatattcataatctccctactttattttcttttatttttaattgat<br>acataatcattatacatatttatgggttaaagtgtaatgttttaatatgtgtacacatattgaccaaatcagggtaattttgcatttgtaa<br>ttttaaaaaatgctttcttcttttaatatacttttttgtttatcttatttctaatactttccctaatctctttctttcagggcaataatgatacaat<br>gtatcatgcctctttgcaccattctaaagaataacagtgataatttctgggttaaggcaatagcaatatctctgcatataaatatttct<br>gcatataaattgtaactgatgtaagaggtttcatattgctaatagcagctacaatccagctaccattctgcttttattttatggttggg<br>ataaggctggattattctgagtccaagctaggcccttttgctaatcatgttcatacctcttatcttcctcccacagCTCCTGG<br>GCAACGTGCTGGTCTGTGTGCTGGCCCATCACTTTGGCAAAGAATTCACCCC<br>ACCAGTGCAGGCTGCCTATCAGAAAGTGGTGGCTGGTGTGGCTAATGCCCT<br>GGCCCACAAGTATCACTAAGCTCGCTTTCTTGCTGTCCAATTTCTATTAAAG<br>GTTCCTTTGTTCCCTAAGTCCAACTACTAAACTGGGGGATATTATGAAGGGC<br>CTTGAGCATCTGGATTCTGCC
5'UTR Frame Shift mutation	GTCAGATCCGCTAGGGATCCCCACAAATGTGGGAGGGCGATAACCACTCGT<br>ACCGAAAGCGTGAGAAGTTACTACAAGCGGTCCTCCCGGCCACCGTACTGT<br>TCCGCTCCCAGAAGCCCCGGGCGGCGGAAGTCGTCACTCTTAAGAAGGGAC<br>GGGGCCCCACGCTGCGCACCCGCGGGTTTGCTATGGTGAGCAAGGGCGAGG<br>AGCTGTTCACCGGGGTGGTGCCCATCCTGGTCGAGCTGGACGGCGACGTAA<br>ACGGCCACAAGTTCAGCGTGTCCGGCGAGGGCGAGGGCGATGCCACCTACG<br>GCAAGCTGACCCTGAAGTTCATCTGCACCACCGGCAAGCTGCCCGTGCCCT<br>GGCCCACCCTCGTGACCACCCTGACCTACGGCGTGCAGTGCTTCAGCCGCTA<br>CCCCGACCACATGAAGCAGCACGACTTCTTCAAGTCC
No mCherry	GACTGCAGCCTCAGGAGATCTGAATTCTCCAGGCGATCTGACGG

Table S7. Primers for PCRs for cloning.

PCR	Primer name	Primer sequence (5’ -> 3’)	T_a (ºC)
mCherry backbone	BglII-mCherry	ACTGCAGCCTCAGGAGATCTTTACTC GTCCATGCCGCCGG	70
	mCherry-EcoRI	CAGATCGCCTGGAGAATTCATGGTG AGCAAGGGCGAGGAG	
Wild-type	BamHI WT	CAGATCCGCTAGGGATCCCCACAAA TGTGGGAGGGCG	62
	β Globin Rev	AAGCTTATCGATGCGGCCGCGGCAG AATCCAGATGCTCAAGG	
ATG -> ACG	BamHI Mut	CAGATCCGCTAGGGATCCCCACAAA CGTGGGAGGGCG	62
	β Globin Rev	AAGCTTATCGATGCGGCCGCGGCAG AATCCAGATGCTCAAGG	
Optimized uORF	BamHI Opt	CAGATCCGCTAGGGATCCCCACAAA TGGGGAGGGCGATAACCACTCGTTA GAAAGCGTGA	62
	β Globin Rev	AAGCTTATCGATGCGGCCGCGGCAG AATCCAGATGCTCAAGG	
Frame Shift	eGFP internal	AGCACGACTTCTTCAAGTCCGCCATG CC	62
	β Globin Rev	AAGCTTATCGATGCGGCCGCGGCAG AATCCAGATGCTCAAGG	

Table S8. Primers for reporter construct sequence verification.

Primer name	Primer sequence (5’ -> 3’)
5’UTR Rev	CATAGCAAACCCGCGGGTGC
SMN 5’UTR	AGAAGCCCCGGGCGGCGGAA
eGFP internal	AGCACGACTTCTTCAAGTCCGCCATGCC
β Glob Fwd ORF	CGGCATGGACGAGCTGTACAAGCTGCTGGTGGTCTACC CTTGGAC
pBI CMV4 Rev	TGGAGATATCGTCGACAAGC
mCherry seq Fwd	AGTGCCACCTGACGTCGGCAG
mCherry seq Rev	ATGTAACGCGGAACTCCATATATG

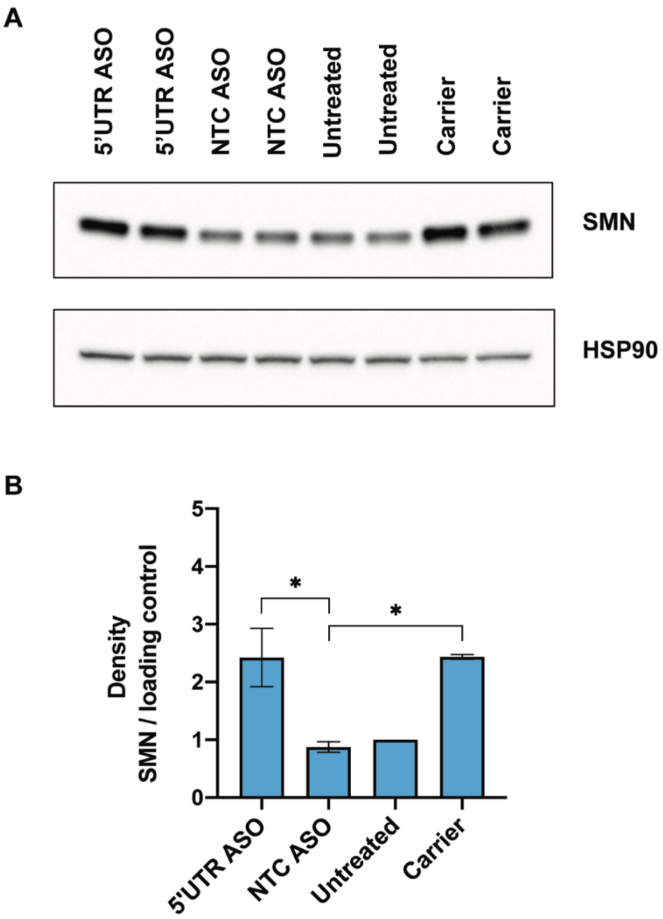

Figure S1. The 5'UTR ASO is effective in a second SMA fibroblast cell line.

A) Immunoblot (15 µg per lane) showing SMN protein levels in GM03813 fibroblasts treated with 600 nM 5'UTR ASO #1 or a non-targeting control (NTC) ASO. B) SMN protein levels normalized to a loading control (alpha tubulin or HSP90) and then calculated as a fold change relative to SMN levels in untreated SMA patient cells. Error bars show SEM. Statistical significance determined by one-way ANOVA followed by Dunnett's test in comparison to NTC ASO. n = 5, except n = 4 for carrier; * $p < 0.02$.

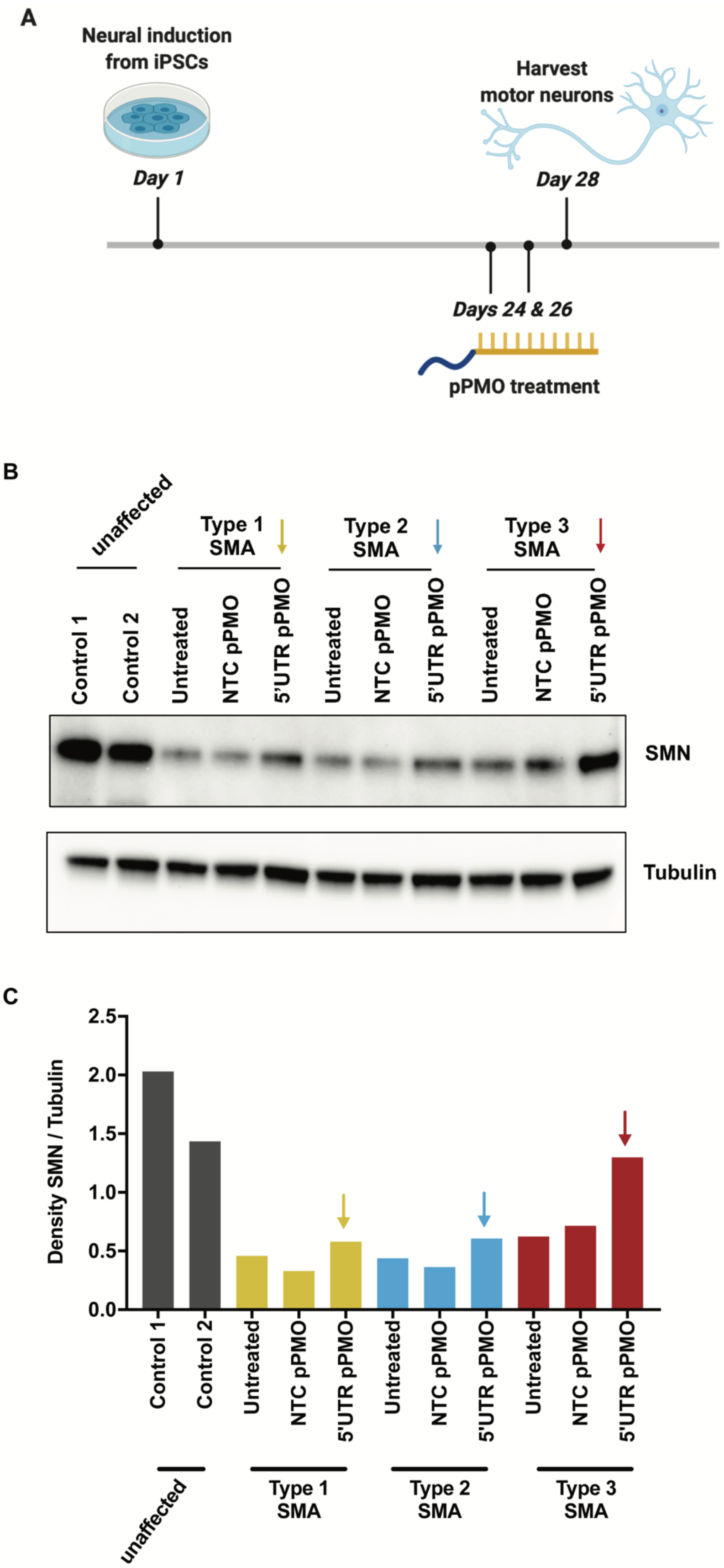
A
Neural induction from iPSCs
Day 1
Harvest motor neurons
Day 28
Days 24 & 26
pPMO treatment
B
unaffected
Type 1 SMA
Type 2 SMA
Type 3 SMA
Control 1
Control 2
Untreated
NTC pPMO
5'UTR pPMO
SMN
Tubulin
C
Density SMN / Tubulin
2.5
2.0
1.5
1.0
0.5
0.0
Control 1
Control 2
Untreated
NTC pPMO
5'UTR pPMO
unaffected
Type 1 SMA
Type 2 SMA
Type 3 SMA

Figure S2. The 5'UTR pPMO increases SMN protein levels in iPSC-derived motor neurons. A) A schematic drawing illustrating timing of treatment and protein extraction in the motor neuron differentiation process. In total, cells were exposed to pPMO for 4 days. B) Immunoblot (4-12% Bis-Tris gel) of motor neuron-like cells (MNs) chemically differentiated from iPSCs derived from control, Type 1 SMA, Type 2 SMA, or Type 3 SMA individuals. MNs were treated with 0.5 μM 5'UTR pPMO or 1 μM NTC pPMO. The NTC pPMO, which was developed for myotonic dystrophy (DM1),[63] targets the CUG expansion in *DMPK* and we did not expect it to change *SMN2* expression. C) Densitometry from immunoblot in panel B. SMN protein levels were normalized to alpha tubulin. n = 1 per cell line.

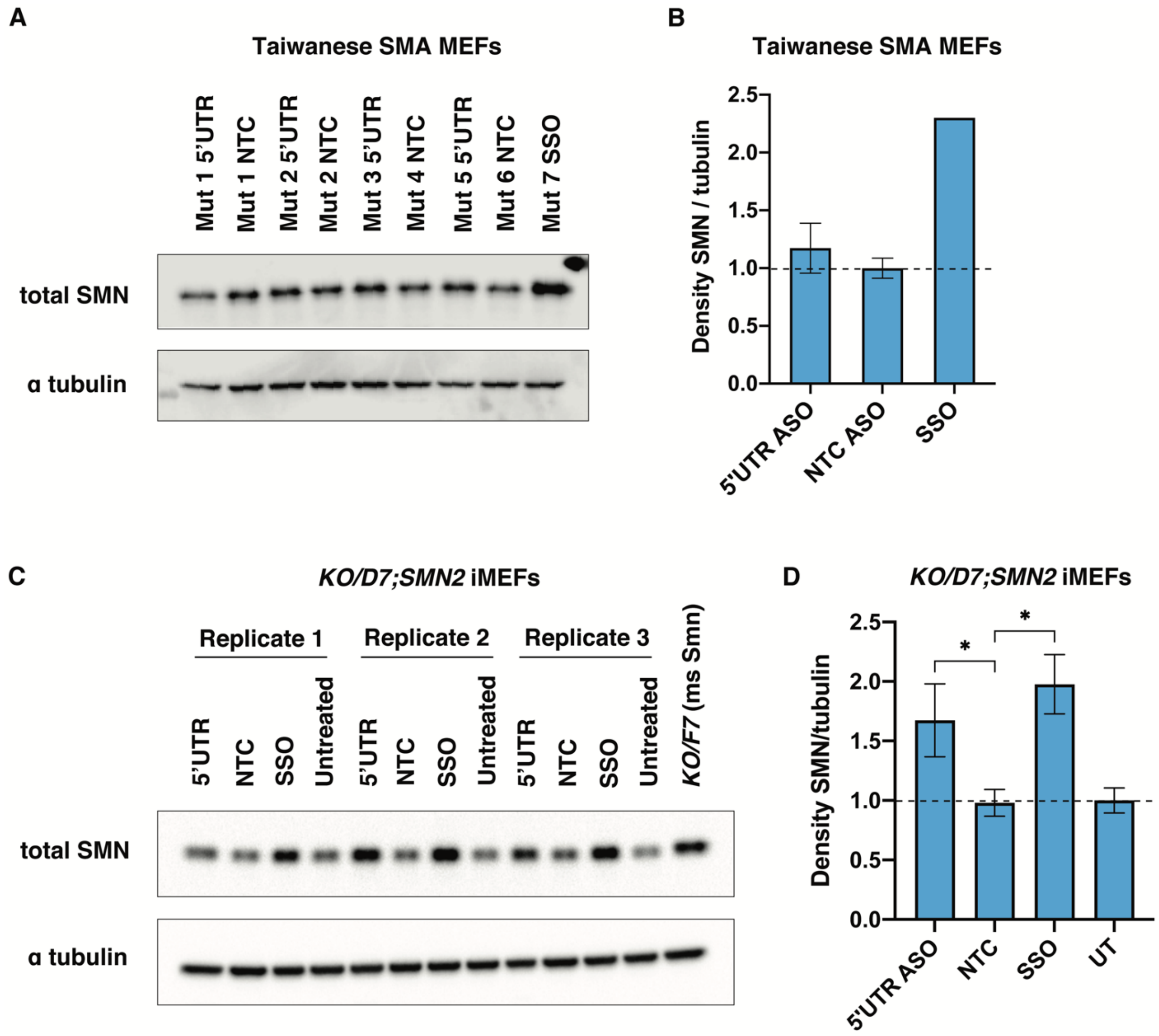

Figure S3. Effect of the 5'UTR ASO in cultured mouse cells.

A) Immunoblot (20 µg per lane) showing SMN protein levels from Taiwanese mouse embryonic fibroblasts treated with 150 nM 2'-MOE ASOs. The SSO (targeting the ISSN1 sequence) was used as a transfection positive control and was in the 2'OMe chemistry. Mut = mutant (SMA genotype). The number in the sample name indicates the embryo from which cells were isolated and grown. B) Densitometry from immunoblot in panel A. SMN protein levels were normalized to alpha tubulin and then calculated as a fold change relative to SMN levels in cells treated with the NTC ASO. Error bars

show propagated error. C) Immunoblot (15 µg per lane) showing SMN protein levels from *KO/D7;SMN2* mouse embryonic fibroblasts[37] treated with 300 nM 2'-MOE ASOs. *KO/F7* has one copy of the mouse Smn gene. D) Densitometry from immunoblot in panel C. SMN protein levels were normalized to alpha tubulin and then calculated as a fold change relative to SMN levels in untreated cells. Error bars show propagated error. * $p < 0.05$.

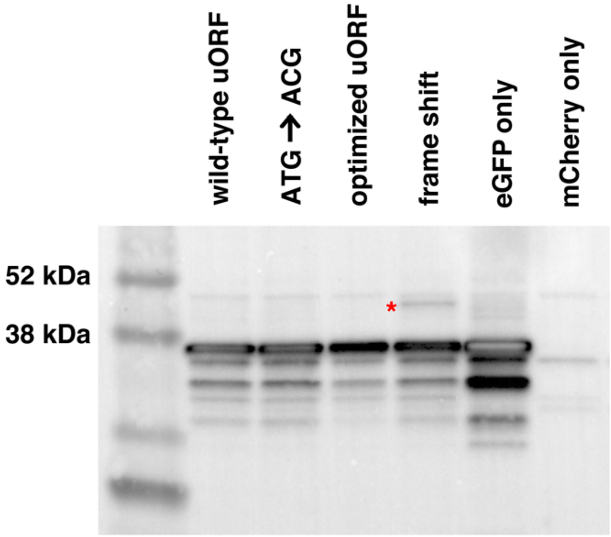

Figure S4. Translation that begins at the upstream start codon is rare (or slow).

The wild-type eGFP fusion protein is 39 kDa. The protein encoded by the frame shift reporter has 53 additional amino acids (encoded by the 5'UTR) and is 45 kDa. This protein is indicated by the red asterisk. The signal from the higher molecular weight protein is visible only after the signal from the canonical protein is saturated.

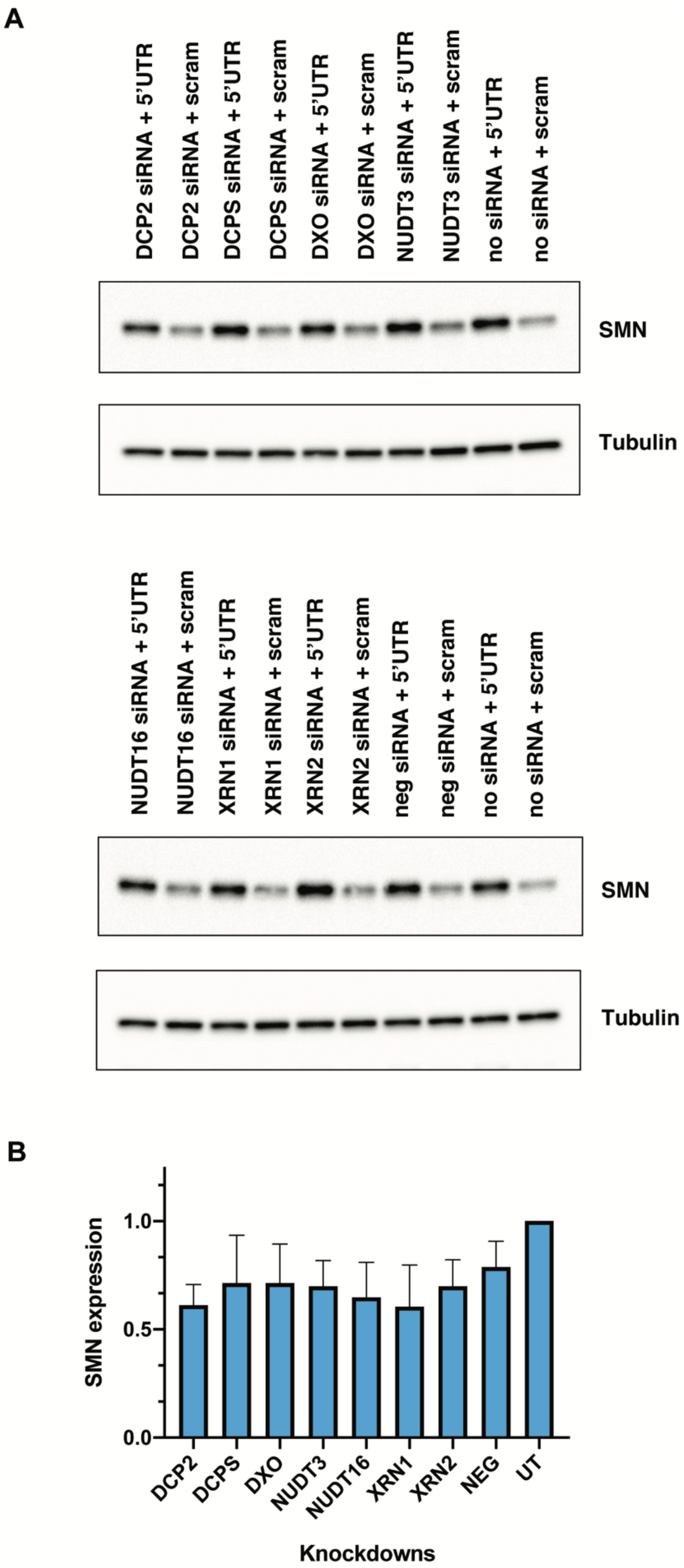
A
DCP2 siRNA + 5'UTR
DCP2 siRNA + scram
DCPS siRNA + 5'UTR
DCPS siRNA + scram
DXO siRNA + 5'UTR
DXO siRNA + scram
NUDT3 siRNA + 5'UTR
NUDT3 siRNA + scram
no siRNA + 5'UTR
no siRNA + scram
SMN
Tubulin
NUDT16 siRNA + 5'UTR
NUDT16 siRNA + scram
XRN1 siRNA + 5'UTR
XRN1 siRNA + scram
XRN2 siRNA + 5'UTR
XRN2 siRNA + scram
neg siRNA + 5'UTR
neg siRNA + scram
no siRNA + 5'UTR
no siRNA + scram
SMN
Tubulin
B
SMN expression
1.0
0.5
0.0
DCP2
DCPS
DXO
NUDT3
NUDT16
XRN1
XRN2
NEG
UT
Knockdowns

Figure S5. Knockdown of decapping factors and exoribonucleases does not abrogate 5'UTR ASO activity. Enzymes involved in RNA decay were knocked down with 100 nM siRNA in GM0232 (SMA) fibroblasts. The next day, the cells were transfected with 300 nM 5'UTR 2'-MOE or scrambled 2'-MOE. A) Immunoblots (15 µg per lane) showing SMN protein levels in SMN-deficient fibroblasts treated with siRNA and ASO combinations indicated. B) SMN protein levels were normalized to alpha tubulin and then calculated as a fold change relative to normalized SMN levels in cells treated with ASO but not siRNA. UT = no siRNA treatment. Error bars show SEM. There was no statistical significance between conditions, as determined by one-way ANOVA followed by Dunnett's test in comparison to NTC. n = 3/4.
